# Large-scale virtual screening of 17,967 IMPPAT phytochemicals identifies multiple CDR1 binders and reveals Apigenin-7-O-glucuronide as a putative CDR1-GSC1 dual-target lead against *Candida albicans* with initial *in-vitro* evidence

**DOI:** 10.3389/fcimb.2026.1940083

**Published:** 2026-09-14

**Authors:** Akshay Kisan Mundhe, Reena Rajkumari

**Affiliations:** Department of Integrative Biology, School of Bio Sciences and Technology, Vellore Institute of Technology, Vellore, Tamil Nadu, India

**Keywords:** ABC transporter, antifungal drug resistance, HR-LCMS, medicinal plants, MM/PBSA, molecular dynamics simulation, natural product drug discovery, β-(1,3)-glucan synthase

## Abstract

The increasing incidence of antifungal resistance in *Candida albicans*, mainly mediated by multidrug efflux transporters and alterations of cell-wall biosynthesis, requires the discovery of new antifungal compounds targeting multiple pathways involved in resistance. In the present study, we employed an integrated computational–experimental workflow to identify potential antifungal phytochemicals from the Indian Medicinal Plants, Phytochemistry and Therapeutics (IMPPAT) database. In the virtual screening, a total of 17,967 phytochemicals were screened against the ATP-binding cassette efflux transporter CDR1, followed by molecular docking, drug-likeness assessment, ADMET filtering, molecular dynamics (MD) simulations, and MM/PBSA binding free-energy calculations. Based on cross-target molecular docking, molecular dynamics simulations, and MM/PBSA analyses of the top ten CDR1 phytochemicals against other antifungal resistance-associated proteins revealed that Apigenin-7-O-glucuronide is the only compound that exhibited favourable interactions with both CDR1 and GSC1, indicating its potential as a putative dual-target inhibitor. Molecular dynamics simulations showed the stability of the protein–ligand interactions, and MM/PBSA analysis confirmed Apigenin-7-O-glucuronide as the most energetically favourable CDR1 inhibitor (ΔGtotal = −35.56 kJ mol−1), and also showed favourable binding with GSC1 (ΔGtotal = −11.69 kJ mol−1). The HR-LCMS analysis of hydroethanolic extract of *Eucalyptus camaldulensis* gave putative evidence of presence of Apigenin-7-O-glucuronide based on accurate mass and database matches. The extract also showed concentration-dependent antifungal activity against *Candida albicans* ATCC 10231 and environmental drug-resistant *Candida* and emerging pathogenic yeast isolates with minimum inhibitory concentration (MIC) of 1.56 mg/mL and minimum fungicidal concentration (MFC) of 6.25 mg/mL. Since the biological evaluation was performed using a crude hydroethanolic extract rather than the purified phytochemical, the observed antifungal activity cannot be exclusively attributed to Apigenin-7-O-glucuronide but provides preliminary biological support for the computational predictions and highlights the need for future validation using the purified compound and target-specific mechanistic studies. Together, these findings identify Apigenin-7-O-glucuronide as a putative dual-target antifungal lead and demonstrate the value of integrating large-scale virtual screening with preliminary biological evaluation for natural product-based antifungal drug discovery.

## 1 Introduction

Fungal infections are an increasing global health problem, affecting more than one billion people annually and causing more than 1.5 million deaths worldwide. The increasing number of immunosuppressed patients such as those undergoing organ transplantation, cancer chemotherapy, long-term corticosteroid treatment, intensive care therapy, and broad-spectrum antibiotic therapy is directly related to the increasing incidence of invasive fungal diseases (Brown et al., 2012a; Brown et al., 2012b; Denning, 2024). The clinical importance of fungal pathogens was further emphasised during the COVID-19 pandemic that was associated with a significant increase in opportunistic fungal infections among hospitalised patients (Ghosh et al., 2021; Nucci et al., 2021). To address this growing burden, the World Health Organization (WHO) published its first fungal priority pathogens list, ranking *Candida albicans* as one of the highest-priority fungal pathogens requiring urgent research and therapeutic development (Jaromin et al., 2024).

*Candida albicans* is an opportunistic dimorphic yeast that asymptomatically colonizes multiple anatomical sites in healthy individuals, but can cause superficial mucosal infections, bloodstream infections and life-threatening invasive candidiasis when host immune defences are compromised (Achkar and Fries, 2010; Ford et al., 2015; Costa-de-Oliveira and Rodrigues, 2020). It is known to possess several virulence factors that contribute to its pathogenicity such as the ability to switch between yeast and hyphae morphological forms, biofilm formation, secretion of hydrolytic enzymes, phenotypic plasticity and the ability to adapt efficiently to different host environments (Jacobsen et al., 2012; Mukaremera et al., 2017). Such adaptive features not only favour the survival of fungi, but also significantly contribute to the reduced susceptibility to antifungal therapies.

Due to their broad spectrum and good clinical safety, azoles remain the most widely prescribed antifungal agents for the treatment of candidiasis. However, the prolonged and indiscriminate use of these agents led to the emergence of multidrug-resistant strains of *Candida albicans* which are increasingly associated with therapeutic failure and recurrent infections (Arendrup and Patterson, 2017). Antifungal resistance in *Candida albicans* is multifactorial, including alterations in ergosterol biosynthesis, target-site mutations, biofilm-associated protection, stress adaptation and active drug efflux. Among these mechanisms, ATP-binding cassette (ABC) transporters play a key role in the active efflux of structurally unrelated antifungal agents from fungal cells, reducing intracellular drug concentrations to sub-therapeutic levels. Drug Resistance Protein 1 (CDR1) is the major ABC efflux transporter responsible for clinical azole resistance (**Figure 1**) and one of the most promising molecular targets to restore antifungal susceptibility (Mundhe and Rajkumari, 2025b).

**Figure 1:**
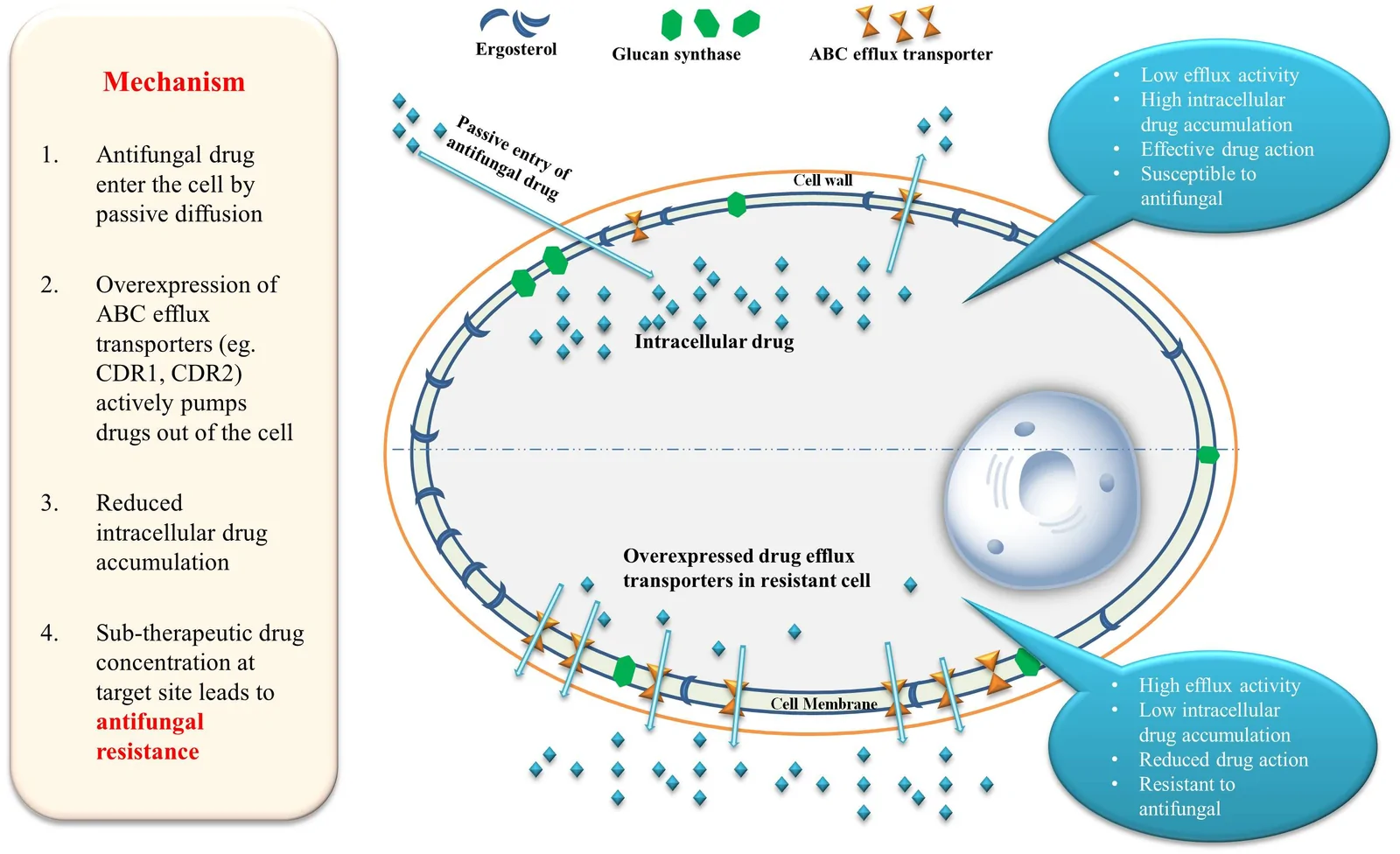
Mechanism of action of ABC transporter CDR1 in antifungal resistance. Overexpression of drug efflux proteins such as CDR1 and CDR2 is a major mechanism of multiple resistance in *Candida albicans* by actively expelling structurally diverse antifungal agents, thereby reducing their efficacy and leading to treatment failure.

Much effort has been dedicated to the discovery of CDR1 inhibitors; however, only a handful of compounds have advanced to clinical applications due to poor selectivity, host toxicity or inappropriate pharmacokinetic properties. Antifungal resistance in *Candida albicans* is coordinated by interconnected molecular pathways and not by a single resistance mechanism. Together with CDR1-mediated drug efflux, proteins involved in ergosterol biosynthesis (ERG1, ERG6 and ERG11), the closely related multidrug transporter CDR2 and the cell wall β-(1,3)-glucan synthase GSC1 contribute to the survival of fungi under antifungal stress (Akins and Sobel, 2017; Mundhe and Rajkumari, 2025b). Therefore, the identification of phytochemicals that can target CDR1 and complementary resistance-associated proteins simultaneously is a promising multitarget strategy to increase antifungal efficacy and potentially reduce the emergence of resistance (Mundhe and Rajkumari, 2025b; Mundhe and Rajkumari, 2025a; Mundhe et al., 2026; Mundhe and Rajkumari, 2026).

Natural products represent an invaluable source of structurally diverse bioactive molecules for antimicrobial drug discovery. They are characterised by a remarkable chemical diversity and often show polypharmacological properties, with the ability to interact with several biological targets (Mundhe and Rajkumari, 2025a). India is one of the richest repositories of medicinal plant biodiversity in the world. Thousands of plant species are widely used in traditional health care systems such as Ayurveda, Siddha and Unani medicine. The Indian Medicinal Plants, Phytochemistry and Therapeutics (IMPPAT) database has tremendously enhanced the potential for natural product-based drug discovery by offering an extensive and curated repository of phytochemicals isolated from Indian medicinal plants (Mohanraj et al., 2018; Vivek-Ananth et al., 2023). Different antifungal activities have been reported for a range of flavonoids, coumarins, phenolic acids, alkaloids, terpenoids and glycosides (Mundhe et al., 2026). For example, baicalein, quercetin, and apigenin have been reported to interfere with ergosterol biosynthesis; thymol, carvacrol, and eugenol disrupt fungal membrane integrity; berberine, curcumin, and geraniol have been shown to modulate ABC efflux transporters and restore azole susceptibility; whereas compounds such as farnesol interfere with biofilm formation, hyphal development, and other virulence-associated pathways (Ahmad et al., 2011; Perlin, 2015b, Perlin, 2015a; Mundhe and Rajkumari, 2025a; Mundhe et al., 2026; Mundhe and Rajkumari, 2026). However, systematic large-scale detailed studies specifically addressing CDR1 inhibition by Indian medicinal plant phytochemicals are still lacking.

Advances in computer-aided drug discovery have recently transformed the early stage of natural product screening by the combination of high-throughput virtual screening, molecular docking, pharmacokinetic prediction, molecular dynamics (MD) simulations and binding free-energy calculations (Mohanraj et al., 2018; Vivek-Ananth et al., 2023). Furthermore, the availability of highly accurate AlphaFold-predicted protein structures has also enabled structure-based drug discovery against fungal proteins lacking experimentally resolved three-dimensional structures. The combination of computational approaches offers a rapid and inexpensive platform for the prioritisation of promising lead molecules prior to experimental evaluation with significant reduction in time and resources needed for conventional natural product screening (Mundhe and Rajkumari, 2025a; Mundhe and Rajkumari, 2026; Mundhe et al., 2026).

Despite these advances, most computational investigations have been limited to relatively small phytochemical libraries or individual antifungal targets, namely ERG11. Few studies have addressed whether phytochemicals predicted to inhibit CDR1 also show multitarget activity against other proteins associated with antifungal resistance, and there is little comprehensive screening of large phytochemical datasets against the multidrug efflux transporter CDR1. Filling this gap could result in the identification of lead compounds that can simultaneously target multiple resistance mechanisms in *Candida albicans*, offering a more effective way to overcome multidrug resistant fungal infections. Although computational methods such as molecular docking and molecular dynamics simulation have become widely accepted for early-stage lead identification, these approaches predict molecular recognition rather than biological inhibition. Therefore, experimental validation remains essential to establish target-specific inhibitory activity.

Therefore, the present study employed an integrated structure-based computational workflow to systematically screen 17,967 phytochemicals from the Indian Medicinal Plants, Phytochemistry and Therapeutics (IMPPAT) database against the multidrug efflux transporter CDR1 of *Candida albicans*. Candidate compounds were prioritized through molecular docking, hydrogen-bond interaction analysis, drug-likeness and ADMET evaluation, followed by 100 ns molecular dynamics simulations and MM/PBSA binding free-energy calculations to evaluate the stability and binding energetics of protein–ligand complexes. Cross-target analyses were subsequently performed to identify phytochemicals with multitarget inhibitory potential against major antifungal resistance-associated proteins. Finally, the computational predictions were complemented with preliminary biological evaluation of fractionated crude extracts putatively containing prioritized phytochemicals to initially support the biological relevance of the identified computational leads.

## 2 Materials and methods

### 2.1 Computational workflow

#### 2.1.1 Overall study design

An integrated computational and experimental workflow was employed to identify phytochemicals with inhibitory potential against the multidrug efflux transporter CDR1 of *Candida albicans*. The workflow involved construction of a phytochemical library from the Indian Medicinal Plants, Phytochemistry and Therapeutics (IMPPAT) database, target protein preparation, molecular docking-based virtual screening, interaction profiling, drug-likeness and ADMET filtering, cross-target analysis against additional antifungal resistance-associated proteins, molecular dynamics (MD) simulation, and MM/PBSA binding free-energy calculations (**Figure 2**). Finally, computational predictions were complemented with initial *in vitro* evaluation using HR-LCMS-guided phytochemical characterization and antifungal assessment of selected medicinal plant extracts.

**Figure 2:**
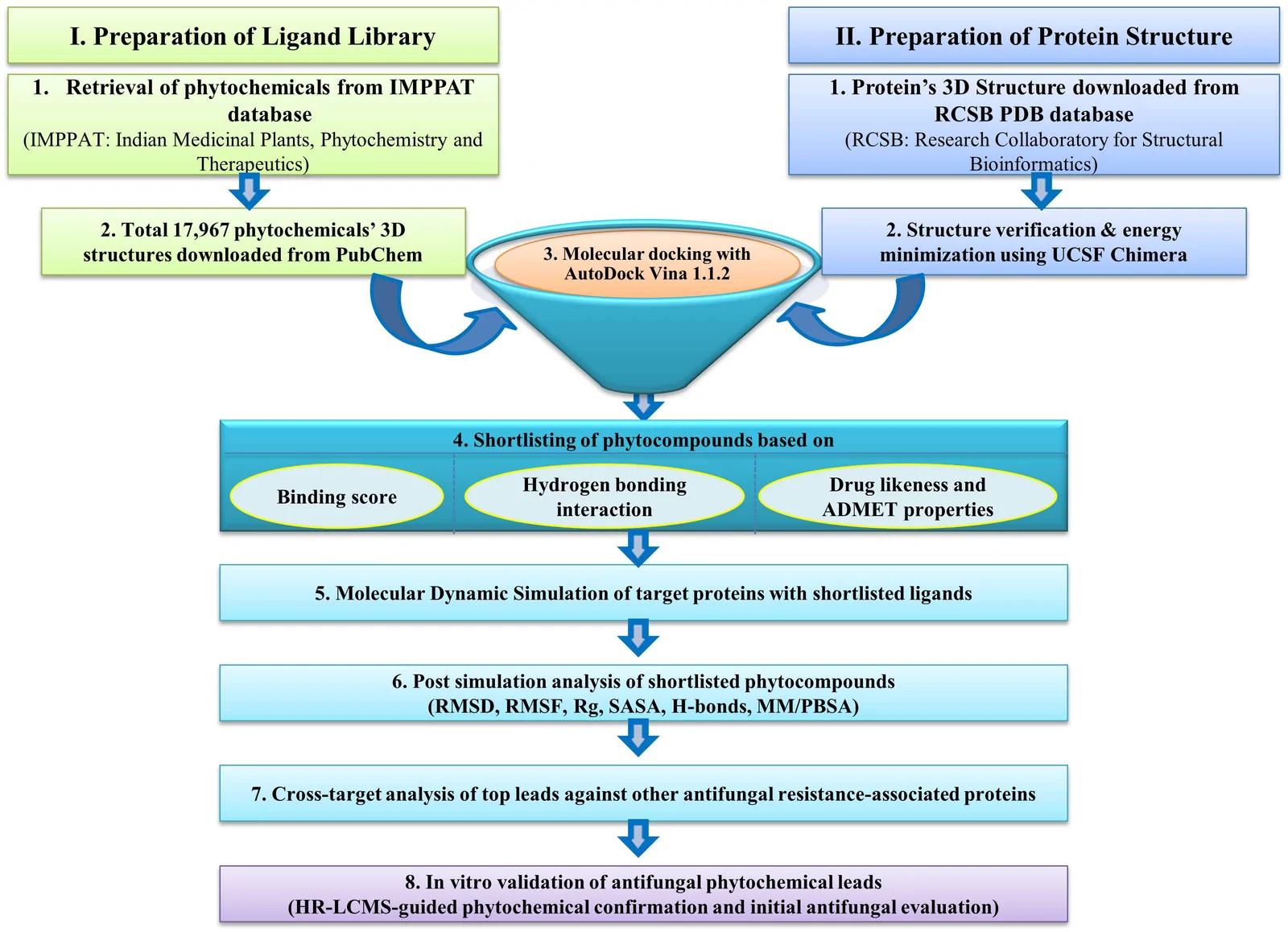
Overall workflow employed for the identification and prioritization of phytochemicals targeting CDR1 and other antifungal resistance-associated proteins in *Candida albicans*.

#### 2.1.2 Phytochemical library preparation

The phytochemical library was constructed using the Indian Medicinal Plants, Phytochemistry and Therapeutics (IMPPAT) database (version 3.0), a manually curated repository integrating phytochemical constituents, medicinal plants, therapeutic applications, and molecular information of Indian medicinal flora (Mohanraj et al., 2018; Vivek-Ananth et al., 2023). IMPPAT 3.0 was selected as the primary source of phytochemicals because it provides a comprehensive collection of structurally characterized natural products suitable for large-scale virtual screening and computer-aided drug discovery (Atanasov et al., 2021; Mohanraj et al., 2018; Vivek-Ananth et al., 2023).

A total of 17,967 phytochemicals were retrieved from the IMPPAT database to construct the ligand library used for computational screening. Three-dimensional molecular structures of the selected phytochemicals were subsequently obtained from the PubChem database (Wang et al., 2009; Kim et al., 2023). The retrieved structures were downloaded in Structure Data File (SDF) format and standardized prior to molecular docking.

Ligand preparation was performed using Open Babel (version 3.1.1) to convert the SDF files into MOL2 format, followed by preparation of Protein Data Bank, Partial Charge, and Atom Type (PDBQT) files using AutoDock Tools scripts. During ligand preparation, hydrogen atoms were added, Gasteiger partial charges were assigned, and rotatable bonds were defined to generate docking-compatible ligand structures for AutoDock Vina (Mundhe and Rajkumari, 2026).

#### 2.1.3 Target protein preparation

The amino acid sequence of the multidrug efflux transporter Candida Drug Resistance Protein 1 (CDR1) from *Candida albicans* SC5314 was retrieved from the UniProtKB database (UniProt: The universal protein knowledgebase in 2023, 2023). Since no experimentally resolved three-dimensional structure of CDR1 was available in the Protein Data Bank (PDB), the predicted structure generated by the AlphaFold Protein Structure Database was used for molecular docking (Jumper et al., 2021; Varadi et al., 2022).

The same procedure was followed for the additional antifungal resistance-associated proteins ERG1, ERG6, CDR2, and GSC1, whereas the experimentally resolved crystal structure of ERG11 was obtained from the Research Collaboratory for Structural Bioinformatics Protein Data Bank (RCSB PDB) (Berman et al., 2000).

All protein structures were imported into UCSF Chimera (version 1.17) for preprocessing prior to docking (Pettersen et al., 2004). Water molecules, non-essential heteroatoms and ligands were removed where applicable, missing hydrogen atoms were added and the protein structures were energy minimised with the AMBER ff14SB force field to remove steric clashes and optimise local geometry. The prepared structures were then exported in PDB format for further analysis.

The stereochemical quality of the protein models was evaluated by PROCHECK through the SAVES v6.0 server, which provided Ramachandran plots for the assessment of the distribution of backbone dihedral angles and overall structural quality (Laskowski et al., 1993; Laskowski et al., 1996). The protein structures with the highest number of residues in the most favoured regions were deemed suitable for molecular docking. Then, the active site residues and ligand binding pockets were predicted using Computed Atlas of Surface Topography of Proteins (CASTp) version 3.0 and the coordinates of the identified binding sites were used for grid box generation during molecular docking (Tian et al., 2018).

#### 2.1.4 Molecular docking

A structure based virtual screening was performed using AutoDock Vina (version 1.2) to identify phytochemicals with potential inhibitory activity against the multidrug efflux transporter CDR1 of Candida albicans. The improved conformational search algorithm and scoring function of AutoDock Vina, delivering better docking accuracy and computational efficiency for large-scale virtual screening, lead to the selection of AutoDock Vina (Trott and Olson, 2010).

Prior to docking, the prepared protein structures and ligand molecules were converted into PDBQT format using AutoDock Tools. Polar hydrogen atoms were retained, Gasteiger partial charges were assigned, and atom types were defined according to AutoDock requirements. Docking calculations were performed using predefined grid boxes centred on the predicted ligand-binding pockets identified by CASTp. The grid centre coordinates and dimensions for each target protein were selected to completely encompass the respective active-site residues and are summarized in **Table 1** (Mundhe and Rajkumari, 2025a; Mundhe and Rajkumari, 2026; Mundhe et al., 2026).

**Table 1:** Configuration used for molecular docking grid parameters used in AutoDock Vina for *Candida albicans* target proteins.

Sr. No.	Target	centre_x	centre_y	centre_z	size_x	size_y	size_z	energy_range	exhaustiveness	numerical mode
1	CDR1	-16.596	1	10.923	80	80	80	3	50	20
2	CDR2	-9.364	-7.255	-6.063	100	100	100	3	50	20
3	ERG1	–5.278	–0.431	–1.159	30	30	30	3	50	20
4	ERG6	6.78	11.047	12.208	30	30	30	3	50	20
5	ERG11	-44.389	-12.556	21.972	70	50	50	3	50	20
6	GSC1	-1.156	9.706	2.033	100	100	100	3	50	20

The exhaustiveness value of 50 was used to increase the conformational sampling and to improve reliability of docking in large-scale virtual screening. The energy range was set to 3 kcal/mol and the maximum of 20 binding poses was generated for each ligand. The lowest binding energy pose was kept for further interaction analysis.

In the first phase, 17967 phytochemicals from the IMPPAT database were screened against CDR1. Ligands were ranked based on predicted binding affinities and top 500 compounds with most favourable docking scores were selected for further analysis. The discovery studio visualiser (version 2024) and pyMOL (version 3.0) were then used to explore the binding orientation and molecular interaction of these compounds. Special attention was given to hydrogen bond interactions with residues in the predicted substrate-binding cavity of CDR1, since hydrogen bonding plays a substantial role in ligand recognition and binding stability.

To improve the reliability of lead selection, compounds exhibiting favourable docking scores but lacking meaningful interactions within the active site were excluded. Candidate phytochemicals were prioritized based on a combination of binding affinity, hydrogen-bond interactions, and binding orientation within the substrate-binding pocket rather than docking score alone. Fewer than 50 phytochemicals satisfied these selection criteria and were subsequently subjected to drug-likeness and ADMET evaluation.

To investigate the possibility of identifying multitarget antifungal agents, the shortlisted CDR1 candidates were further docked against additional antifungal resistance-associated proteins, including ERG1, ERG6, ERG11, CDR2, and GSC1, using the same docking protocol and corresponding grid parameters. Phytochemicals demonstrating favourable binding affinities and conserved interaction profiles across multiple targets were prioritized for subsequent molecular dynamics simulations and MM/PBSA binding free-energy calculations.

#### 2.1.5 Lead prioritization based on protein–ligand interactions, drug-likeness and ADMET evaluation

The phytochemicals selected after molecular docking were further filtered by a multi-parameter screening strategy comprising of protein-ligand interaction, drug-likeness and absorption, distribution, metabolism, excretion and toxicity (ADMET) prediction. This integrated approach was employed to identify compounds with favourable binding characteristics and acceptable pharmacokinetic properties for subsequent molecular dynamics simulations.

The top 500 phytochemicals obtained from molecular docking against CDR1 were first screened by visual inspection using BIOVIA Discovery Studio Visualiser 2024 and PyMOL (version 3.0). Protein–ligand interactions, such as hydrogen bonds, hydrophobic interactions, π–π stacking, π–alkyl interactions, van der Waals interactions, and electrostatic contacts, were analysed. Special attention was given to the formation of hydrogen bonds with amino acid residues present in the predicted substrate-binding cavity of CDR1, as such interactions are important for ligand recognition and stability of the complex. Compounds with good docking scores but without significant interactions on the active site were excluded from further analysis. Instead of using only docking score, we selected candidate phytochemicals based on a combination of binding affinity, binding orientation and interaction profile.

The shortlisted phytochemicals were then screened for their physicochemical properties and drug-likeness using SwissADME web server (Daina et al., 2017). Predicted molecular descriptors were molecular weight, lipophilicity (LogP), hydrogen bond donors, hydrogen bond acceptors, topological polar surface area (TPSA), number of rotatable bonds, gastrointestinal (GI) absorption, blood–brain barrier (BBB) permeability, water solubility, bioavailability score and compliance with Lipinski’s Rule of Five (Lipinski, 2001). Phytochemicals satisfying Lipinski’s criteria or exhibiting only minor deviations while maintaining favourable pharmacokinetic characteristics were retained for subsequent analyses.

To further evaluate the pharmacokinetic and toxicity profiles of the selected phytochemicals, ADMET properties were predicted using ADMETlab 3.0. Parameters including human intestinal absorption, plasma protein binding, cytochrome P450 enzyme inhibition, P-glycoprotein substrate probability, hepatotoxicity, carcinogenicity, mutagenicity, and acute oral toxicity were considered during compound prioritization. Preference was given to phytochemicals demonstrating favourable ADMET profiles together with acceptable drug-likeness characteristics and predicted oral bioavailability.

Following this sequential filtering strategy, fewer than 50 phytochemicals were identified as high-confidence CDR1 candidates. These compounds were subsequently evaluated against additional antifungal resistance-associated proteins (ERG1, ERG6, ERG11, CDR2, and GSC1) to identify phytochemicals with multitarget inhibitory potential. The highest-ranking multitarget candidates were finally selected for molecular dynamics simulations and MM/PBSA binding free-energy calculations.

#### 2.1.6 Cross target analysis

Following lead prioritization based on molecular docking, protein–ligand interactions, and ADMET evaluation, the selected CDR1 phytochemicals were further investigated for their ability to interact with additional antifungal resistance-associated proteins. Cross-target analysis was performed against ERG1, ERG6, ERG11, CDR2, and GSC1 using the same molecular docking protocol, protein preparation procedure, and docking parameters described in Sections 2.1.3–2.1.5 to ensure methodological consistency.

The shortlisted phytochemicals were comparatively evaluated for predicted binding affinity, interaction profile and hydrogen-bond formation in the active-site cavities of each target protein. Special attention was paid to the identification of compounds able to interact with CDR1 and other proteins related to antifungal resistance at same time, possessing a multitarget inhibitory potential. The phytochemicals that exhibited favourable binding characteristics on multiple targets were further subjected to molecular dynamics simulations and MM/PBSA binding free-energy calculations. This approach led to the identification of multitarget lead candidates for subsequent computational and experimental assessment.

#### 2.1.7 Molecular dynamics simulation

Molecular Dynamics Simulations The dynamic stability of the chosen protein–ligand complexes was assessed under physiological conditions using 100 ns molecular dynamics (MD) simulations with GROMACS version 2022.5 (Abraham et al., 2015). MD simulations were performed for selected CDR1 lead compounds and for prioritized multitarget complexes identified during cross-target analysis, including the GSC1–Apigenin-7-O-glucuronide complex.

The CHARMM36 all-atom force field was used to generate the protein topology files and ligand topology and parameter files were generated using the CHARMM General Force Field (CGenFF). The protein-ligand complexes were solvated in a cubic simulation box of TIP3P water molecules with a minimum distance of 1.0 nm between the protein surface and the box boundaries. To keep the system neutral, appropriate Na+ or Cl- ions were added to compensate for the net charge of each simulation system (Vanommeslaeghe et al., 2010; Huang and MacKerell Jr, 2013).

Prior to the production simulation, all systems were energy minimised using the steepest descent algorithm until the maximum force converged below the default GROMACS cutoff. Then the minimised systems were equilibrated for 100 ps at 300 K under constant number of particles, volume and temperature (NVT) conditions, followed by constant number of particles, pressure and temperature (NPT) equilibration for 100 ps at 1 bar pressure. Temperature was maintained at constant using the modified Berendsen (V-rescale) thermostat and pressure coupling was done using Parrinello-Rahman barostat. The long range electrostatic interactions were calculated with the Particle Mesh Ewald (PME) method. All covalent bonds to hydrogen atoms were constrained with the LINCS algorithm, allowing a simulation time step of 2 fs (Essmann and Geiger, 1995; Bussi et al., 2007).

After equilibration, for each selected protein-ligand complex 100 ns of production MD simulations were carried out under periodic boundary conditions. Trajectory coordinates were sampled at regular time intervals and analysed with built-in GROMACS utilities. The root mean square deviation (RMSD), root mean square fluctuation (RMSF), radius of gyration (Rg), solvent accessible surface area (SASA) and intermolecular hydrogen bond analyses were done to evaluate the structural stability and conformational behaviour of each complex. These parameters were used to evaluate the stability of ligand binding, protein flexibility, structural compactness, solvent exposure and the persistence of the protein-ligand interactions during the simulation period. Additionally, the equilibrated MD trajectories obtained in this study were used for MM/PBSA binding free-energy calculations to further assess the binding affinity of the prioritised phytochemicals.

#### 2.1.8 Binding free energy analysis by MM/PBSA

To further evaluate the binding affinity of the shortlisted phytochemicals, Molecular Mechanics/Poisson–Boltzmann Surface Area (MM/PBSA) calculations were performed on representative trajectories of the 100 ns molecular dynamics simulations. MM/PBSA is a popular end-point free energy method which combines molecular mechanics and continuum solvent models to predict protein-ligand binding affinities with relatively low computational cost (Kollman et al., 2000; Kumari et al., 2014; Genheden and Ryde, 2015).

The binding free energy (ΔG_binding) was calculated according to:


$$\text{ΔG}\_\text{binding }=\text{ G}\_\text{complex }-\;\left(\text{G}\_\text{protein }+\text{ G}\_\text{ligand}\right)$$

Where G_complex, G_protein and G_ligand are the free energies of the protein-ligand complex, the free protein and the free ligand respectively. Total binding free energy was calculated with van der Waals, electrostatic, polar solvation and non-polar solvation energy.

More negative ΔG_binding values indicated stronger and energetically favourable protein-ligand interactions. The MM/PBSA results were interpreted together with molecular docking scores, protein-ligand interaction analyses, and MD trajectory parameters, including RMSD, RMSF, radius of gyration (Rg), solvent-accessible surface area (SASA), hydrogen-bond occupancy, and principal component analysis (PCA), to prioritize phytochemicals exhibiting stable binding and favourable interaction energetics (Kumari et al., 2014) (Genheden and Ryde, 2015; Wang et al., 2019). Binding free energies are presented as mean ± standard deviation (SD) obtained from 201 trajectory frames generated by gmx_MMPBSA.

### 2.2 Experimental workflow

#### 2.2.1 Plant material and phytochemical extraction

Medicinal plants were selected based on the occurrence of phytochemicals prioritized during the computational screening. Plant materials were authenticated at the Plant Anatomy Research Centre (PARC), Chennai, and different plant parts were collected according to the reported distribution of the target phytochemicals. After authentication, the plant materials were washed thoroughly, shade-dried under ambient conditions to preserve heat-sensitive phytochemicals, and pulverized into a fine powder using a mechanical grinder (**Supplementary Figure 1**) (Dai and Mumper, 2010; Azwanida, 2015).

The extraction strategy was guided by the physicochemical properties of the computationally prioritized phytochemicals, which were predominantly polar or moderately polar. Accordingly, 70% ethanol, 70% methanol, and distilled water were employed as extraction solvents (Dai and Mumper, 2010; Azmir et al., 2013). Following preliminary optimization, ultrasound-assisted extraction (UAE) was selected because of its higher extraction efficiency and reduced extraction time compared with conventional maceration. Briefly, 25 g of powdered plant material was mixed with 200 mL of extraction solvent (1:8, w/v) and extracted using an ultrasonic bath operating at 40 kHz for 30 min with intermittent sonication cycles (**Figure 3**) (25 s on/5 s off) (Tiwari, 2015; Chemat et al., 2017).

**Figure 3:**
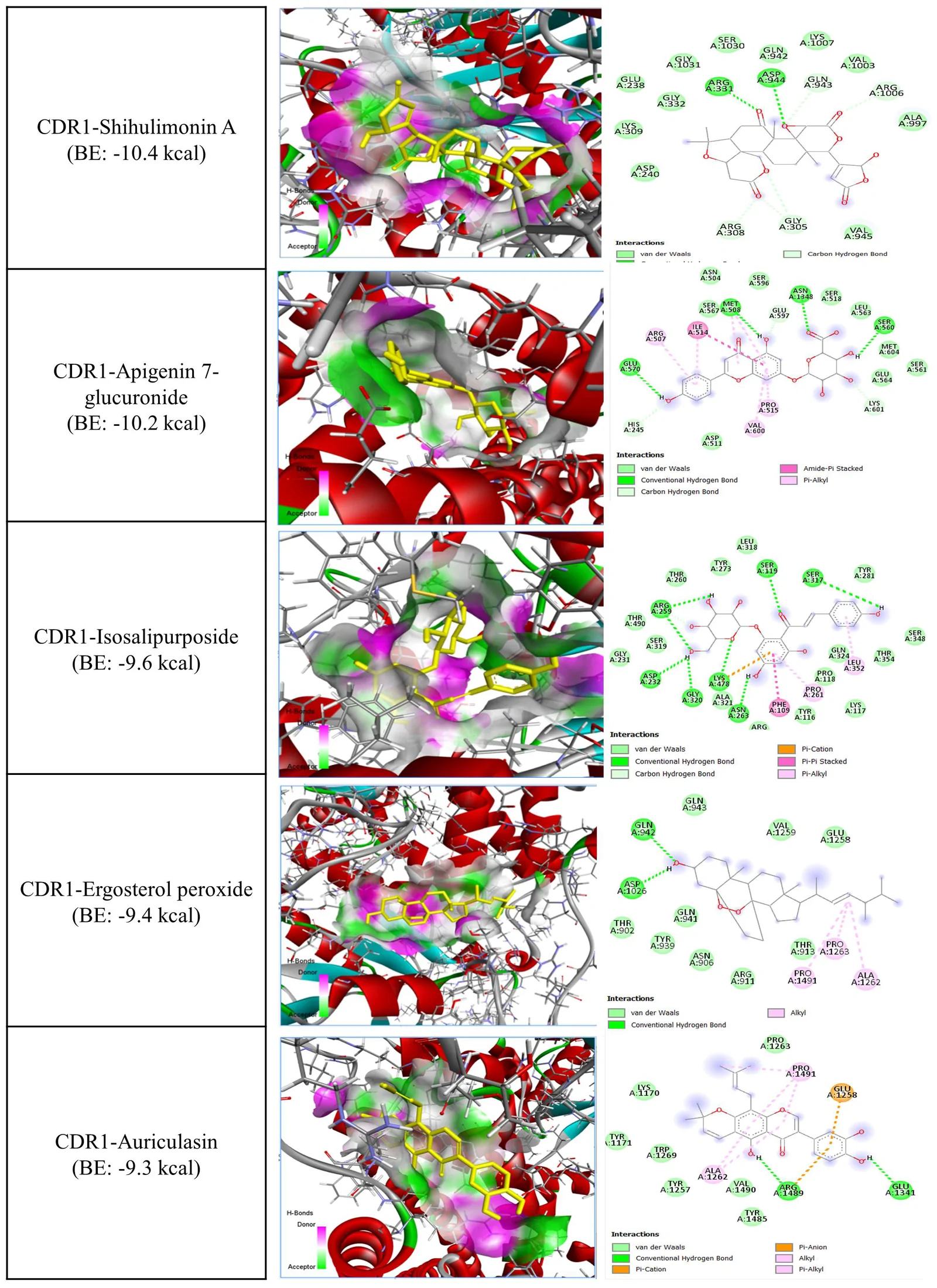
Three-dimensional binding poses and two-dimensional interaction maps of the five highest-ranked phytochemicals docked within the CDR1 substrate-binding pocket.

The extracts were filtered, centrifuged at 6000 rpm for 6 min, concentrated under reduced pressure using a rotary evaporator, and dried under aseptic conditions. The resulting crude extracts were stored at 4 °C until HR-LCMS characterization and antifungal susceptibility testing (**Supplementary Figure 1**).

#### 2.2.2 HR-LCMS characterization

The phytochemical composition of the crude extracts was characterized by high-resolution liquid chromatography–mass spectrometry (HR-LCMS) at the Central Instrumentation Facility, Vellore Institute of Technology, Vellore. Dried extracts were dissolved in analytical-grade methanol and analysed by HR-LCMS in positive ionization mode to obtain high-resolution chromatographic and mass spectral profiles (Wolfender et al., 2019; Khare et al., 2020).

Compound annotation was performed according to accurate mass and database matching only. Therefore, phytochemical identities should be regarded as putative identification rather than definitive structural confirmation. Accurate m/z values, molecular ion information and comparison with available phytochemical databases and published reports (Wolfender et al., 2019; Kumar et al., 2022) were used to perform compound annotation. Special attention was given to the validation of phytochemicals screened by molecular docking, interaction analysis, molecular dynamics simulation and MM/PBSA calculations, thus providing analytical support for the phytochemical-guided plant selection strategy prior to biological evaluation.

#### 2.2.3 Qualitative antifungal assay

Preliminary antifungal activity of the selected plant extracts was evaluated by the agar well diffusion method using *Candida albicans* ATCC 10231 and representative environmental yeast isolates. Fresh cultures grown on Sabouraud Dextrose Agar (SDA) were adjusted to a 0.5 McFarland standard (approximately 1–5 × 10^6^ CFU/mL) and uniformly inoculated onto SDA plates to produce a confluent lawn.

Wells of 6 mm diameter were prepared using a sterile cork borer and filled with the respective plant extract concentrations. For *Eucalyptus camaldulensis*, the extract was tested at 100, 50, 25, and 12.5 mg/mL, whereas *Glycyrrhiza glabra* was evaluated using additional two-fold dilutions down to 1.56 mg/mL. For *Silybum marianum*, the hydroethanolic extract was evaluated in combination with fluconazole, beginning with 50 mg/mL extract and 50 μg/mL fluconazole, followed by two-fold serial dilutions. Solvent controls served as negative controls and fluconazole as the positive control. All assays were performed in triplicate. After incubation at 37 °C for 24 h, antifungal activity was assessed by measuring the diameter of the inhibition zones surrounding each well.

#### 2.2.4 Quantitative antifungal assay

The antifungal activity of the selected plant extracts was further quantified using the broth microdilution method according to the Clinical and Laboratory Standards Institute (CLSI) M27 guidelines. For quantitative assays, the standardized 0.5 McFarland inoculum was diluted 100-fold to obtain a final inoculum density of approximately 1–5 × 10^4^ CFU/mL (Carvalhaes et al., 2022).

Two-fold serial dilutions of each extract were prepared in sterile 96-well microtiter plates. Each well contained 100 μL of double-strength Mueller–Hinton broth, 100 μL of the corresponding plant extract dilution, and 10 μL of yeast inoculum. Following incubation at 37 °C for 24 h, 10 μL of resazurin solution (0.1 mg/mL) was added as a viability indicator. The minimum inhibitory concentration (MIC) was defined as the lowest extract concentration that prevented the colour change of resazurin from blue to pink, indicating inhibition of fungal growth.

For determination of the minimum fungicidal concentration (MFC), aliquots from wells showing complete growth inhibition were subcultured onto fresh SDA plates and incubated at 37 °C for 24–48 h. The lowest concentration that produced no visible colony growth following subculture was recorded as the MFC.

## 3 Results

### 3.1 Preparation and validation of target protein structures

To enable structure-based virtual screening, three-dimensional models of six antifungal resistance-associated proteins of *Candida albicans*, namely CDR1, CDR2, ERG1, ERG6, ERG11, and GSC1, were prepared and validated prior to molecular docking. Since experimentally resolved crystal structures were unavailable for most targets, high-confidence structural models predicted by AlphaFold were retrieved and processed for subsequent computational analyses. The prepared protein structures are illustrated in **Supplementary Figure 2**.

The stereochemical quality of the protein models was evaluated using PROCHECK through Ramachandran plot analysis. As shown in **Supplementary Figure 3**, the majority of amino acid residues for all six proteins were located within the most favoured regions, indicating good structural quality suitable for molecular docking and molecular dynamics simulations. Among the selected targets, ERG6 exhibited the highest percentage of residues in the most favoured regions (94.3%), followed by CDR2 (91.7%), ERG1 (91.6%), and CDR1 (91.4%), whereas GSC1 (89.9%) and ERG11 (86.3%) also demonstrated acceptable stereochemical quality for computational analysis.

The Ramachandran statistics validated the good structural integrity and stereochemical reliability of the protein models selected for the subsequent stages of active site prediction, molecular docking, molecular dynamics simulations and MM/PBSA binding free-energy calculations.

### 3.2 Active-site prediction and large-scale virtual screening identify multiple putative CDR1 inhibitors

The ligand-binding pocket of CDR1 was predicted using CASTp and used to define the docking region for large-scale virtual screening. The predicted active-site cavity comprised residues lining the substrate-binding pocket of the transporter and served as the basis for grid box generation during molecular docking. Similar active-site prediction was performed for the remaining antifungal resistance-associated proteins to facilitate subsequent cross-target analysis (values depicted in **Table 1**).

Virtual screening of 17,967 phytochemicals retrieved from the IMPPAT database against the CDR1 binding pocket identified a diverse set of compounds with favourable predicted binding affinities. Top 500 phytochemicals shortlisted on the basis of docking scores were analysed for detailed interactions. Further investigation of protein-ligand interactions revealed that 33 phytochemicals formed one or multiple hydrogen bonds with residues in the predicted CDR1 active site. Lipinski’s Rule of Five and pharmacokinetic filtering were applied to further reduce the dataset to 10 lead phytochemicals chosen for full computational evaluation (**Table 2**).

**Table 2:** Top 10 phytochemicals shortlisted following molecular docking, hydrogen-bond interaction analysis, and Lipinski filtering against CDR1.

Sr. No.	Pubchem ID	Phytochemical name	No. of H bonds with a. a. residues	Binding energy	ml. wt.	Source plant
1	5321283	Shihulimonin A	2	-10.4	502.52	*Euodia fraxinifolia*
2	5319484	Apigenin 7-glucuronide	4	-10.2	446.36	*Cardiospermum halicacabum, Eucalyptus camaldulensis,Gmelina arborea, Gmelina asiatica*
3	5318659	Isosalipurposide	8	-9.6	434.4	*Acacia dealbata, Asystasia gangetica, Rungia repens*
4	5351516	Ergosterol peroxide	2	-9.4	428.66	*Ananas comosus, Arenaria kansuensis, Broussonetia papyrifera, Glycine max, Oryza sativa, Rhynchosia minima, Solanum dulcamara*
5	5358846	Auriculasin	2	-9.3	420.46	*Maclura cochinchinensis, Maclura pomifera,Millettia extensa*
6	5318547	Isodemethylwedelolactone	1	-9.1	300.22	*Eclipta prostrata*
7	5319492	Rhynchosin	2	-9	302.24	*Rhynchosia beddomei*
8	5321494	Undulatoside A	6	-8.8	354.31	*Tecomella undulata*
9	5319891	Moracin H	1	-9.1	338.36	*Morus alba, Discladium squarrosum*
10	5319924	Cudraflavone C	3	-9.1	422.48	*Maclura pomifera*

The most promising compounds in the list were Shihulimonin A with the highest predicted binding affinity to CDR1 (−10.4 kcal/mol) followed by Apigenin 7-O-glucuronide (−10.2 kcal/mol) and Isosalipurposide (−9.6 kcal/mol). Other promising phytochemicals were Ergosterol peroxide, Auriculasin, Isodemethylwedelolactone, Rhynchosin, Undulatoside A, Moracin H and Cudraflavone C with favourable docking scores along with interactions with key residues in the predicted substrate-binding cavity (**Table 2**).

Representative three-dimensional binding poses and two-dimensional interaction maps of the ten lead phytochemicals are presented in **Figures 3**, **4**. The interaction profiles revealed that these compounds established multiple hydrogen-bond, hydrophobic, and π-mediated interactions with residues located within the CDR1 binding pocket, supporting their potential to interfere with substrate recognition and efflux function. To provide a comparative benchmark, the docking pose and interaction profile of the reference inhibitor Beauvericin are shown in **Figure 5**.

**Figure 4:**
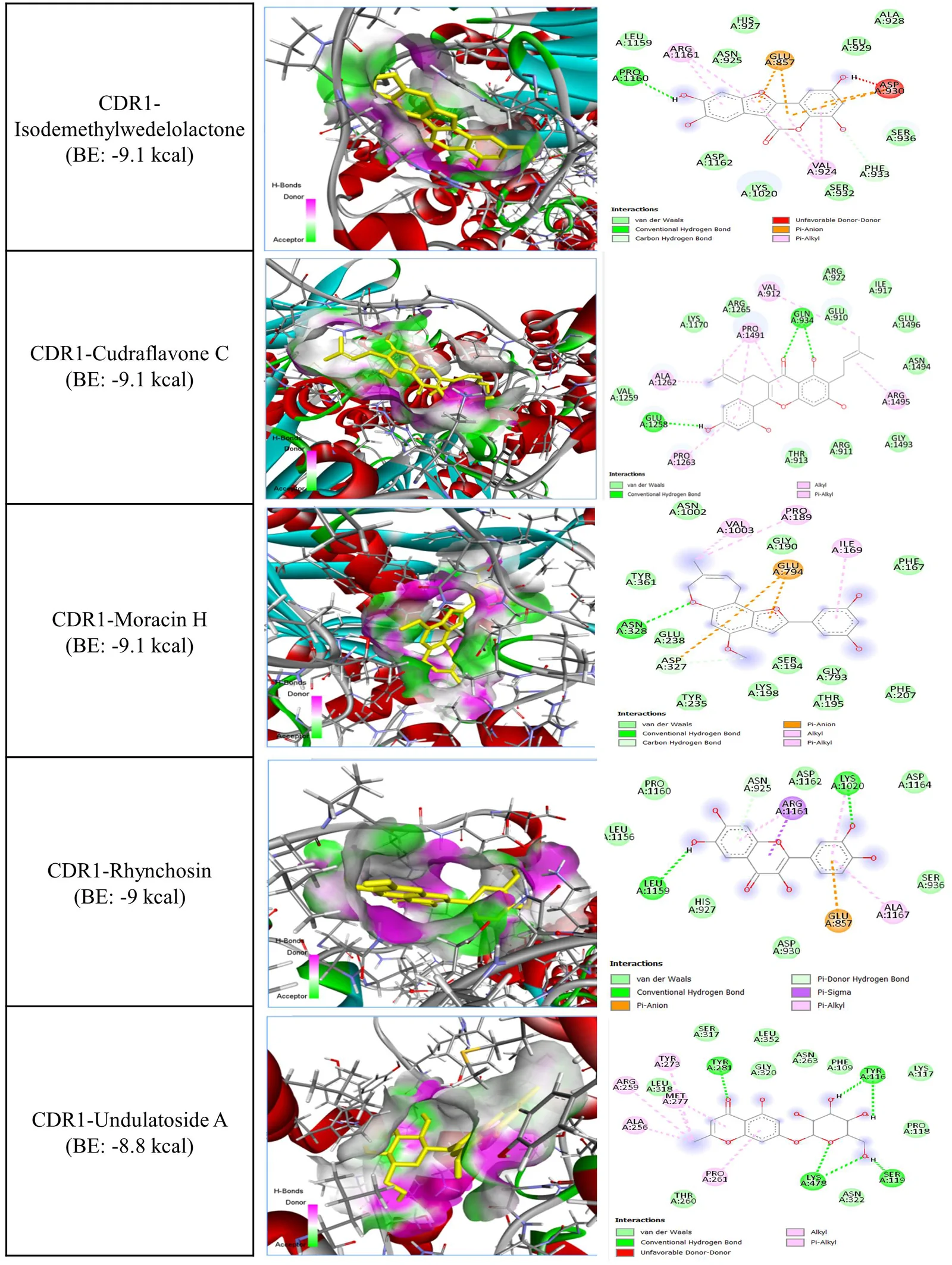
Three-dimensional binding poses and two-dimensional interaction maps of the remaining shortlisted phytochemicals interacting with the CDR1 binding pocket.

**Figure 5:**
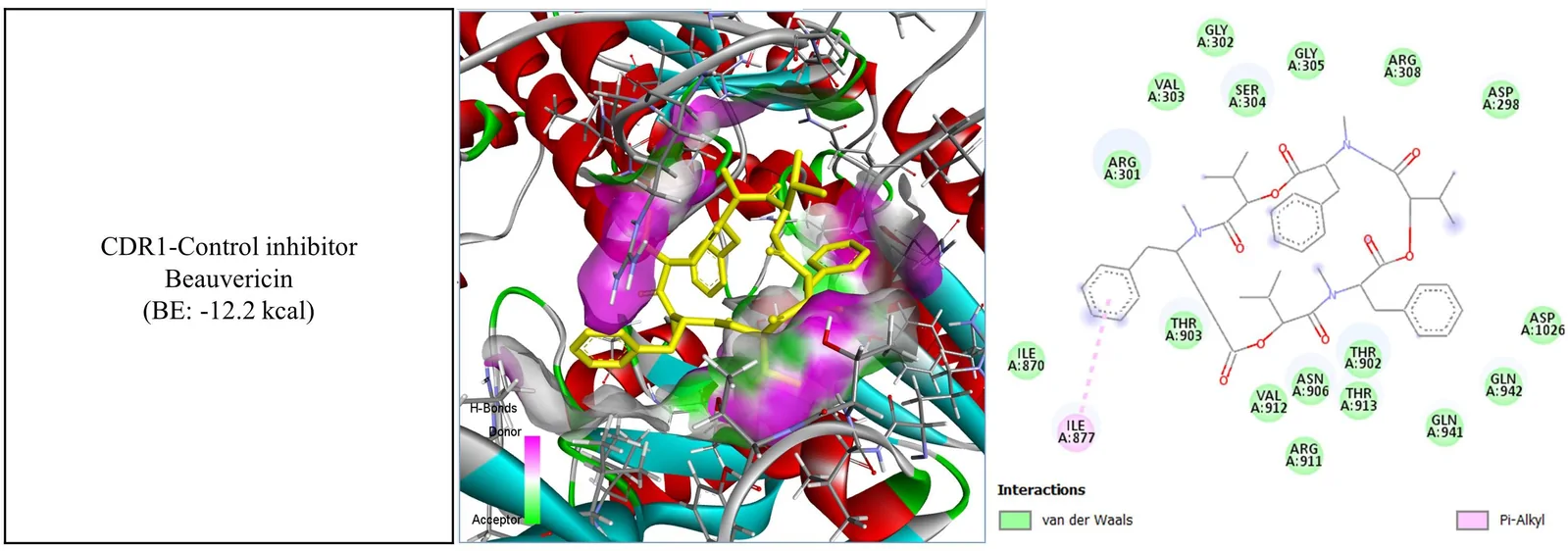
Three-dimensional binding pose and two-dimensional interaction map of the reference inhibitor Beauvericin within the CDR1 substrate-binding pocket.

### 3.3 Cross-target analysis identifies Apigenin-7-O-glucuronide as a putative CDR1–GSC1 dual-target lead

To investigate whether the prioritized CDR1 lead compounds possessed broader antifungal potential, all ten lead phytochemicals identified through large-scale virtual screening were subsequently docked against five additional antifungal resistance-associated targets, namely CDR2, ERG1, ERG6, ERG11, and GSC1, using the same molecular docking protocol. This cross-target analysis aimed to identify compounds capable of simultaneously targeting multiple resistance mechanisms in *Candida albicans*.

Among the ten shortlisted phytochemicals, Apigenin-7-*O*-glucuronide was the only compound that demonstrated favourable binding affinity toward an additional resistance-associated protein. In addition to its high affinity for the CDR1 substrate-binding pocket (−10.2 kcal/mol), Apigenin-7-*O*-glucuronide also exhibited strong interaction with GSC1, with a docking score of −9.5 kcal/mol, whereas the remaining lead phytochemicals did not demonstrate comparable binding with the additional protein targets.

The three-dimensional docking poses further demonstrated that Apigenin-7-*O*-glucuronide occupied well-defined binding cavities in both proteins (**Figure 6**). Within CDR1, the ligand established multiple hydrogen-bond and hydrophobic interactions involving residues including Glu570, Ser567, Ser596, Asn1348, Met604, Glu564, and Arg507, together with π-mediated interactions involving Ile514, contributing to stable accommodation within the substrate-binding cavity. Similarly, docking against GSC1 revealed extensive interactions with residues such as Asp1451, Asp1568, Thr1419, Gln1411, Ser1415, Lys1700, Arg1696, and Phe1418, supporting favourable ligand recognition within the catalytic binding region.

**Figure 6:**
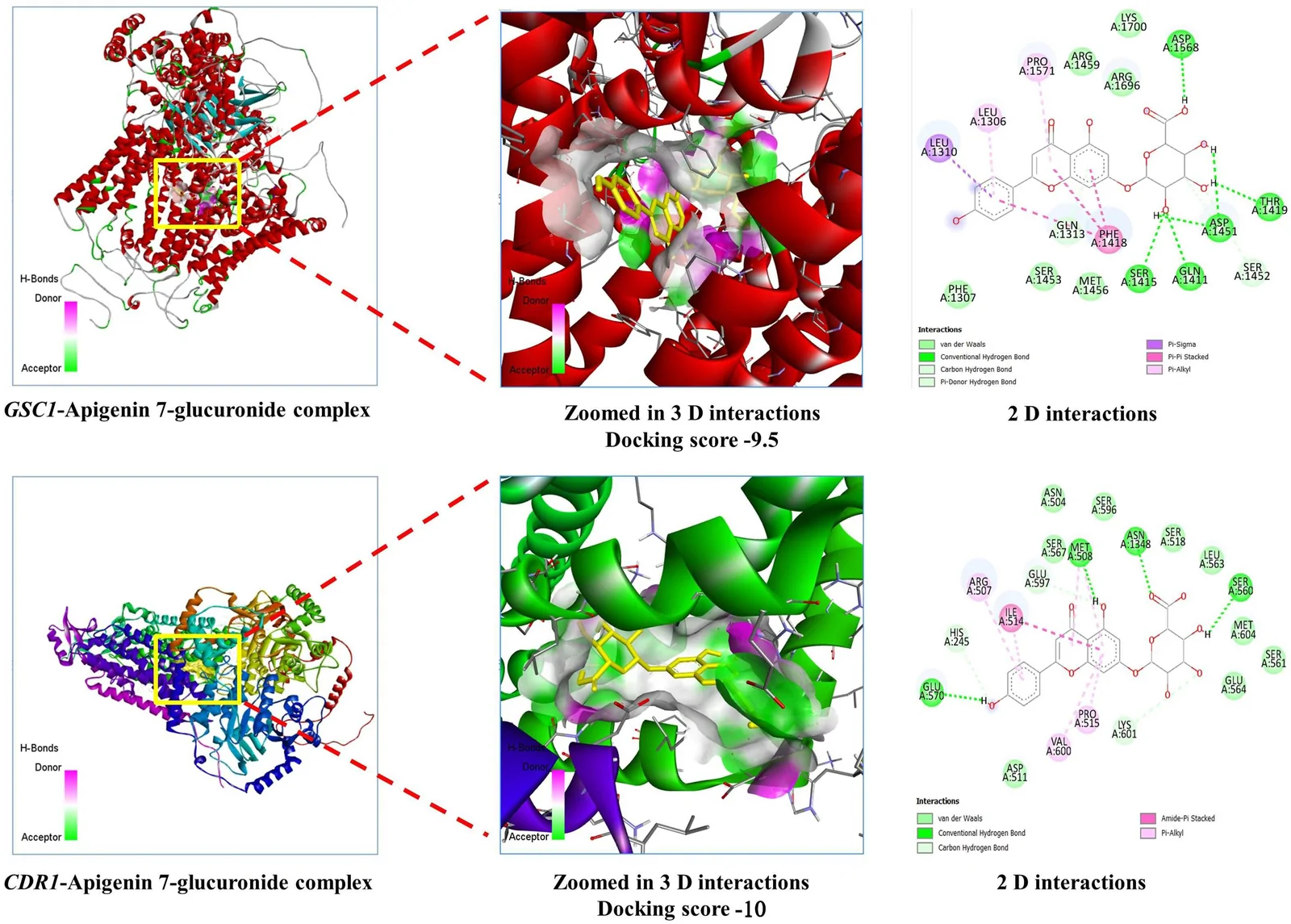
Molecular docking interactions of Apigenin-7-*O*-glucuronide with the antifungal resistance-associated targets CDR1 and GSC1 of *Candida albicans*.

The ability of Apigenin-7-*O*-glucuronide to interact with both the drug-efflux transporter CDR1 and the β-1,3-glucan synthase GSC1 suggests a potential dual-target mechanism capable of simultaneously interfering with antifungal drug efflux and fungal cell-wall biosynthesis. Consequently, this phytochemical was selected for subsequent comparative molecular dynamics simulations and binding free-energy analyses to further investigate the stability of its interactions with both target proteins.

### 3.4 Molecular dynamics simulations reveal stable binding of the top CDR1 lead phytochemicals

To investigate the dynamic stability of the top-ranked CDR1 lead compounds identified through molecular docking, 100 ns MD simulations were performed for all ten CDR1–ligand complexes. Structural stability, residue flexibility, protein compactness, solvent accessibility, conformational dynamics, and intermolecular hydrogen-bond persistence were evaluated using RMSD, RMSF, radius of gyration (Rg), solvent-accessible surface area (SASA), principal component analysis (PCA), and hydrogen-bond occupancy analyses, respectively.

#### 3.4.1 Structural stability of CDR1–ligand complexes

The RMSD trajectories demonstrated that all protein–ligand complexes remained stable throughout the 100 ns simulation, although differences in conformational behaviour were observed among individual phytochemicals (**Figure 7**). The native CDR1 protein equilibrated at approximately 0.8–1.2 nm, providing a reference for comparison.

**Figure 7:**
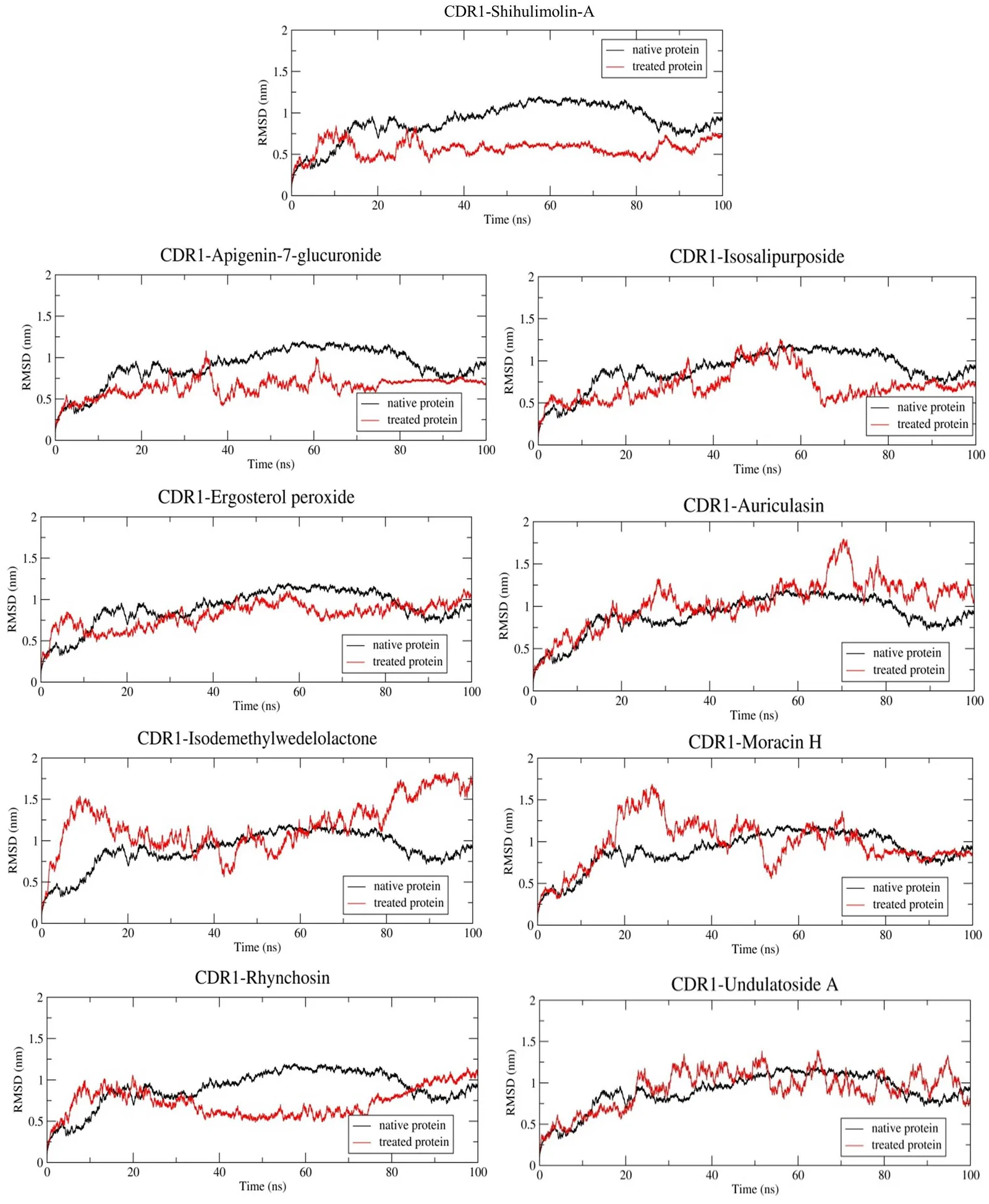
RMSD analysis of CDR1–phytochemical complexes during 100 ns molecular dynamics simulations. Root mean square deviation (RMSD) of the native CDR1 protein (black) and ligand bound CDR1 complexes (red) during 100 ns molecular dynamics simulation. 552 RMSD was calculated throughout the entire simulation to verify the structural stability of the 553 protein-ligand complexes in relation to the starting minimized structure.

Among the evaluated compounds, Shihulimonin A, Apigenin-7-*O*-glucuronide, and Rhynchosin exhibited the most stable trajectories, with RMSD values largely maintained between 0.5 and 0.8 nm following initial equilibration. These complexes showed only minor fluctuations throughout the simulation, indicating stable accommodation of the ligands within the CDR1 binding pocket. Ergosterol peroxide also demonstrated a relatively stable trajectory, fluctuating around 0.8–1.0 nm during the latter half of the simulation.

Isosalipurposide displayed a transient increase in RMSD to approximately 1.2 nm between 45 and 60 ns, after which the complex returned to a stable conformation comparable to the native protein. In contrast, Auriculasin, Isodemethylwedelolactone, and Moracin H exhibited comparatively larger structural deviations during portions of the simulation, reaching RMSD values of approximately 1.5–1.8 nm before stabilizing. Undulatoside A maintained intermediate stability, with fluctuations predominantly within 0.8–1.2 nm throughout the simulation. Importantly, none of the complexes showed abrupt increases in RMSD indicative of ligand dissociation or structural collapse, demonstrating that all shortlisted phytochemicals remained associated with the CDR1 binding cavity during the simulation.

Then the flexibility was analysed residue-wise by RMSF analysis (**Supplementary Figure 4**). All complexes displayed similar fluctuation profiles with pronounced mobility at the N-terminal residues where RMSF values reached approximately 2.5–4.0 nm, reflecting the intrinsic flexibility of terminal regions. Fluctuations of the majority of the residues were less than 0.5 nm, except for these terminal segments, indicating preservation of the overall protein architecture. However, a consistent localised increase in flexibility was observed around residues 780–830, corresponding to a solvent exposed loop region rather than the predicted ligand-binding pocket. Importantly, no significant increase in RMSF was observed within the residues directly involved in ligand binding, indicating that the interaction of the shortlisted phytochemicals did not induce local destabilisation of the functional binding site.

#### 3.4.2 Protein compactness and solvent accessibility

The radius of gyration (Rg) (**Supplementary Figure 5**) was used to evaluate the structural compactness of the protein–ligand complexes. The native protein’s average Rg was around 3.9–4.0 nm for the entire simulation. Relatively small changes around this value observed for the complexes with Apigenin-7-O-glucuronide, Shihulimonin A, Ergosterol peroxide and Rhynchosin suggest that the global protein fold was maintained. Auriculasin and Isodemethylwedelolactone, contrasted, showed short-lived peaks near 4.3-4.4 nm, indicative of a moderate conformational expansion throughout the simulation time, without any evidence of structural unfolding. The other phytochemicals showed intermediate behaviour with fluctuations mostly below 4.2 nm, suggesting that the protein maintained its structure during the simulation.

Consistent with the Rg results, SASA analysis showed only moderate changes in solvent accessibility during the simulation (**Supplementary Figure 6**). The SASA values of the native CDR1 protein were mostly between 675 and 700 nm², whereas ligand-bound complexes were mostly fluctuating between 685 and 720 nm². The SASA profiles were relatively stable for Apigenin-7-O-glucuronide and Ergosterol peroxide, showing only small oscillations throughout the simulation which suggests less alteration in solvent exposure. Whereas, Auriculasin, Moracin H, Rhynchosin and Undulatoside A showed greater variations in SASA, indicating local conformational changes and not global structural instability. To summarise, the analyses of Rg and SASA showed that the ligand binding did not detrimentally impact the structural compactness and solvent accessibility of CDR1.

#### 3.4.3 Conformational dynamics and persistence of hydrogen-bond

Differences in conformational sampling between the simulated complexes were also distinguishable by principal component analysis (PCA) (**Figure 8**; **Supplementary Figure 7**). Shihulimonin A, Apigenin-7-O-glucuronide, Ergosterol peroxide, Auriculasin, Isodemethylwedelolactone, Moracin H and Rhynchosin occupied relatively compact conformational clusters indicating restricted large scale motions and stable dynamic behaviour during the simulation.

**Figure 8:**
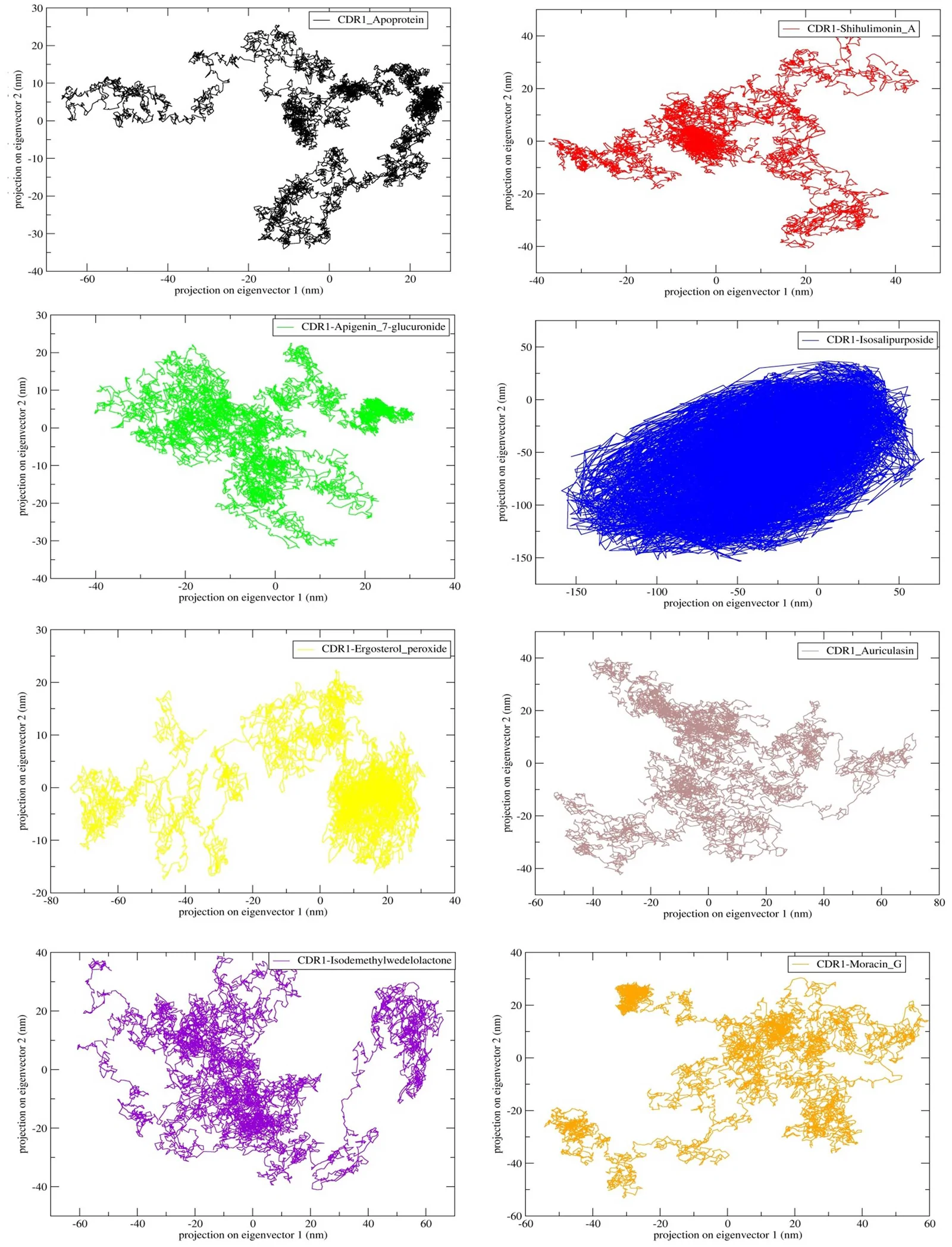
Principal component analysis (PCA) of CDR1–phytochemical complexes. Projection of molecular dynamics trajectories onto the first two principal components (PC1 and PC2) for native CDR1 and complexes with Shihulimonin A, Apigenin-7-*O*-glucuronide, Isosalipurposide, Ergosterol peroxide, Auriculasin, Isodemethylwedelolactone, and Moracin H. PCA was performed to evaluate large-scale collective motions and conformational sampling during the simulations.

The persistence of intermolecular interactions was subsequently evaluated by monitoring hydrogen-bond occupancy over the 100 ns trajectories (**Figure 9**). Shihulimonin A exhibited a constant hydrogen bond occupancy of 4–6 bonds, with temporary rises to 7–8 interactions, signifying persistent ligand anchoring throughout the entire simulation. Initially, apigenin-7-O-glucuronide formed 6–8 hydrogen bonds and stabilised at around 3–5 hydrogen bonds at 30–35 ns, indicating adaptation to a stable binding conformation. The number of hydrogen bonds between isosalipurposide and ergosterol peroxide was maintained at the level of 4–7 and 4–6 with small fluctuations during most of the simulation. Rhynchosin also exhibited persistent interaction, keeping approximately 5–7 hydrogen bonds throughout the trajectory.

**Figure 9:**
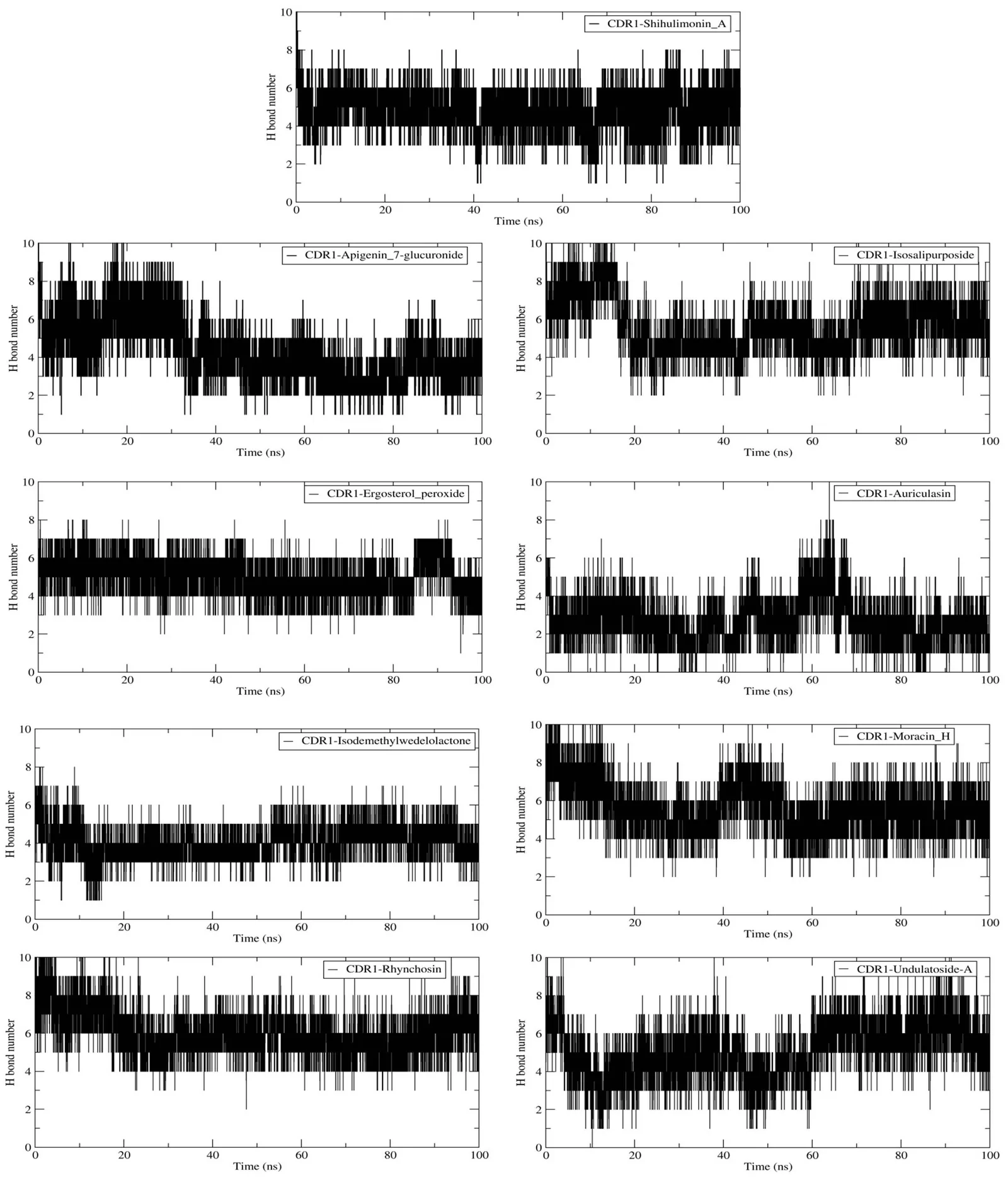
Hydrogen-bond occupancy analysis of CDR1–phytochemical complexes. Time-dependent hydrogen-bond occupancy between CDR1 and the ten shortlisted phytochemicals throughout the 100 ns molecular dynamics simulations. The number of intermolecular hydrogen bonds was monitored to evaluate the persistence and stability of ligand–protein interactions over the simulation period.

Among the rest of the compounds, Auriculasin exhibited comparatively less number of interactions with a general 2–4 hydrogen bonds, only transiently increased to 7–8 hydrogen bonds around 60–65 ns. Isodemethylwedelolactone showed relatively constant interaction through 3–5 hydrogen bonds, while Moracin H showed a decline from about 7–9 hydrogen bonds during the early stages of the simulation to 4–6 hydrogen bonds after equilibration. Undulatoside A fluctuated between 3 and 6 hydrogen bonds, with increased interaction occupancy observed after approximately 60 ns.

Collectively, the molecular dynamics analyses demonstrated that 9 out of ten phytochemicals identified through large-scale virtual screening formed stable complexes with CDR1 under dynamic conditions. Cudraflavone_C-CDR1 complex was failed in molecular dynamic simulation steps on multiple trials. While several compounds exhibited favourable structural stability, all nine CDR1 lead phytochemicals were subsequently subjected to cross-target docking against CDR2, ERG1, ERG6, ERG11, and GSC1 to investigate their multitarget potential. Among these candidates, Apigenin-7-*O*-glucuronide was the only phytochemical that additionally exhibited favourable binding toward GSC1 (Section 3.3), warranting further comparative molecular dynamics simulations against both CDR1 and GSC1. This integrated computational workflow enabled the identification of Apigenin-7-*O*-glucuronide as the most promising putative dual-target antifungal lead for subsequent analyses.

### 3.5 Comparative molecular dynamics of Apigenin-7-*O*-glucuronide with CDR1 and GSC1

To further investigate the multitarget potential of Apigenin-7-*O*-glucuronide, comparative molecular dynamics simulations were performed for its complexes with CDR1 and GSC1. The dynamic behaviour of both protein–ligand systems was evaluated using RMSD, RMSF, radius of gyration (Rg), solvent-accessible surface area (SASA), and intermolecular hydrogen-bond analyses over a 100 ns simulation period (**Figure 10**).

**Figure 10:**
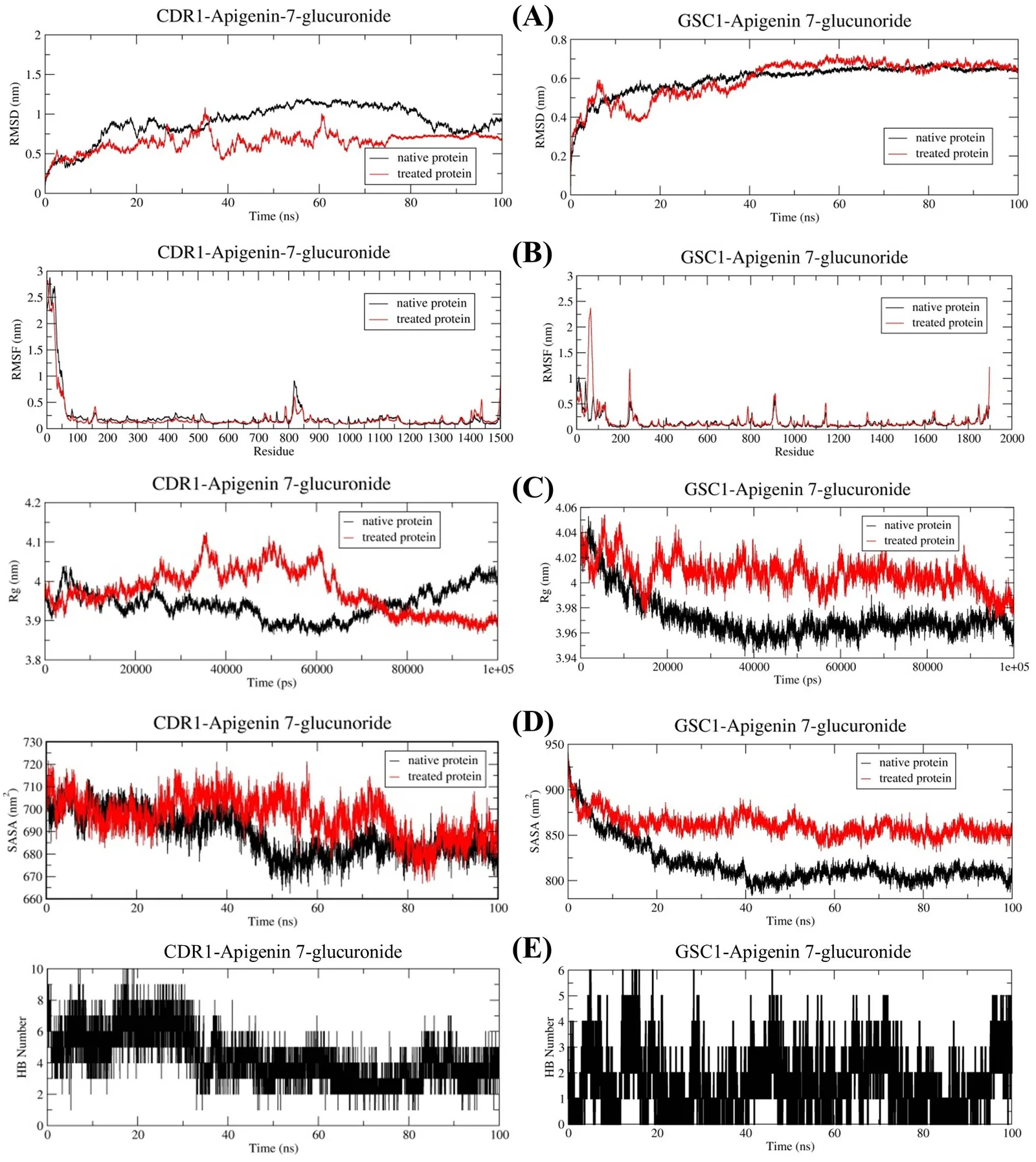
Comparative molecular dynamics study of CDR1 and GSC1 complexed with Apigenin-7-O-glucuronide. *Note:* Comparison of molecular dynamics for CDR1 and GSC1 bound to Apigenin-7-O-glucuronide for 100 ns simulations. **(A)** Root mean square deviation (RMSD), **(B)** root mean square Running Title fluctuation (RMSF), **(C)** radius of gyration (Rg), **(D)** solvent accessible surface area (SASA) and **(E)** intermolecular hydrogen-bond occupancy. Comparative analyses were carried out to assess the dynamic stability of Apigenin-7-O-glucuronide with both antifungal resistance-associated targets.

The RMSD trajectories indicated that both the protein–ligand complexes achieved structural equilibrium in the early stages of the simulation and remained stable thereafter (**Figure 10A**). The CDR1–Apigenin-7-O-glucuronide complex was stabilised with RMSD values mostly in the range of 0.5–0.8 nm. The GSC1 complex was equilibrated at 0.6–0.7 nm with lower fluctuations during the simulation. For both complexes, no abrupt structural deviations were observed, indicating that the ligands were stably accommodated in their respective binding cavities.

The residue-wise flexibility calculated using the RMSF analysis showed similar dynamics for the two proteins (**Figure 10B**). Increased fluctuations were mainly localised to terminal regions and flexible surface loops, as seen in the CDR1 complexes, whereas residues of the ligand-binding pockets showed relatively low mobility. Several loop regions of the GSC1 complex showed localised fluctuations, but the catalytic binding residues were structurally stable during the simulation, suggesting that ligand binding did not induce a significant perturbation of the active site architecture.

The radius of gyration (Rg) profiles showed that both complexes retained compact protein structures during the simulation (**Figure 10C**). The Rg values of the CDR1 complex range from 3.9–4.1 nm, and the GSC1 complex showed a very consistent Rg value of 3.98–4.04 nm, which indicated that the global structural compactness was well-maintained. Neither target showed any signs of protein unfolding or large-scale conformational expansion.

Like the SASA analysis, the solvent accessibility of both protein–ligand systems was relatively stable during the 100 ns simulations (**Figure 10D**). The CDR1 complex had minor variations from 680 to 710 nm2 while the GSC1 complex had SASA values of 800–870 nm2, indicating changes in protein size rather than ligand induced instability. In both cases, the solvent exposure was found to be relatively constant over the trajectories, supporting stable protein conformations after ligand binding.

Analysis of intermolecular hydrogen-bond occupancy further confirmed persistent interactions between apigenin-7-O-glucuronide and both target proteins (**Figure 10E**). The CDR1 complex started with ~6–8 hydrogen bonds that gradually stabilised to 3–5 interactions during the later stages of the simulation. Conversely, the GSC1 complex consistently engaged in 2–5 hydrogen bonds throughout the simulation, with occasional transient increases to six interactions. Both the protein ligand systems displayed a continuous contact of the intermolecular contact over the whole period of the simulation which revealed the stable retention of the ligand in their respective binding sites but the hydrogen bond occupancy was slightly higher in the CDR1 complex.

The comparative molecular dynamics analyses revealed that Apigenin-7-O- glucuronide formed stable complexes with the CDR1 efflux transporter and GSC1 β-1,3- glucan synthase with favourable structural stability, preserved protein compactness, limited residue fluctuations and sustained intermolecular interactions during the entire simulation. These results are consistent with the molecular docking results and provide dynamic evidence for the potential of Apigenin-7-O-glucuronide as a putative dual-target antifungal lead that can simultaneously target fungal drug efflux and cell-wall biosynthesis pathways.

### 3.6 MM/PBSA binding free-energy analysis

To further validate the binding stability of the shortlisted phytochemicals, MM/PBSA binding free-energy calculations were performed using representative trajectories extracted from the 100 ns molecular dynamics simulations. The total binding free energy (ΔGTOTAL) together with the van der Waals (ΔVDWAALS), electrostatic (ΔEEEL), gas-phase (ΔGGAS), and solvation (ΔGSOLV) energy components were calculated to estimate the energetic favourability of ligand binding toward CDR1.

Among the evaluated phytochemicals, Apigenin-7-*O*-glucuronide exhibited the most favourable average MM/PBSA binding free energy toward CDR1 with a ΔGTOTAL of −35.56 kJ/mol, followed closely by Isodemethylwedelolactone (−34.22 kJ/mol). Isosalipurposide (−27.64 kJ/mol), Undulatoside A (−26.48 kJ/mol), and Auriculasin (−25.05 kJ/mol) also demonstrated favourable binding energetics, whereas Moracin H, Ergosterol peroxide, and Rhynchosin exhibited comparatively weaker but still favourable binding affinities (**Table 3A**). In contrast, Shihulimonin A, despite producing the best molecular docking score, yielded a slightly positive binding free energy (+1.31 kJ/mol) after molecular dynamics refinement, indicating that its initially favourable docking pose was not energetically maintained under dynamic simulation conditions.

**Table 3:** MM/PBSA binding free energy calculation.

A. Binding free-energy analysis of the shortlisted phytochemicals against CDR1.						
Sr. No.	Ligand	ΔVDWAALS (kJ/mol)	ΔEEEL (kJ/mol)	ΔGGAS (kJ/mol)	ΔGSOLV (kJ/mol)	ΔGTOTAL (Mean ± SD) (kJ/mol)
1	Apigenin-7-*O*-glucuronide	−47.12	−59.09	−106.21	70.65	−35.56 ± 4.47
2	Isodemethylwedelolactone	−35.53	−25.58	−61.11	26.89	−34.22 ± 3.98
3	Isosalipurposide	−45.59	−28.62	−74.21	46.57	−27.64 ± 2.52
4	Undulatoside A	−35.86	−29.88	−65.74	39.26	−26.48 ± 2.63
5	Auriculasin	−43.42	−5.17	−48.59	23.54	−25.05 ± 2.34
6	Moracin H	−30.79	−8.60	−39.38	17.88	−21.51 ± 2.02
7	Ergosterol peroxide	−37.15	−1.20	−38.36	20.43	−17.92 ± 2.04
8	Rhynchosin	−22.73	−26.68	−49.41	37.37	−12.03 ± 3.38
9	Shihulimonin A	−22.14	−14.25	−36.39	37.7	1.31 ± 2.16
B. Comparative binding free-energy analysis of Apigenin-7-O-glucuronide against CDR1 and GSC1.						
Target protein	Ligand	ΔVDWAALS (kJ/mol)	ΔEEEL (kJ/mol)	ΔGGAS (kJ/mol)	ΔGSOLV (kJ/mol)	ΔGTOTAL (Mean ± SD) (kJ/mol)
CDR1	Apigenin-7-*O*-glucuronide	−47.12	−59.09	−106.21	70.65	−35.56 ± 4.47
GSC1	Apigenin-7-*O*-glucuronide	−24.62	−16.49	−41.10	29.41	−11.69 ± 2.85

Analysis of the individual energy components revealed that van der Waals and electrostatic interactions were the principal contributors to ligand binding. Apigenin-7-O-glucuronide exhibited the strongest van der Waals (−47.12 kJ/mol) and electrostatic (−59.09 kJ/mol) interaction energies among all phytochemicals, resulting in the most favourable gas-phase interaction energy (ΔGGAS = −106.21 kJ/mol). Although positive solvation energies opposed complex formation, these unfavourable contributions were compensated by strong intermolecular interactions, resulting in an overall negative binding free energy for the majority of the shortlisted compounds.

Since Apigenin-7-*O*-glucuronide was identified as the only phytochemical exhibiting favourable binding toward both CDR1 and GSC1 during cross-target analysis, its energetic stability with GSC1 was further investigated using MM/PBSA calculations (**Table 3B**). The GSC1–Apigenin-7-*O*-glucuronide complex exhibited a ΔGTOTAL of −11.69 kJ/mol, indicating a favourable, although comparatively weaker, binding affinity than that observed for CDR1. Similar to the CDR1 complex, binding was predominantly driven by favourable van der Waals (−24.62 kJ/mol) and electrostatic (−16.49 kJ/mol) interactions, which compensated for the unfavourable solvation energy (29.41 kJ/mol).

Hence, the MM/PBSA analyses refined the prioritization of the shortlisted phytochemicals by incorporating dynamic interaction energetics. While molecular docking initially ranked Shihulimonin A as the strongest CDR1 binder, subsequent molecular dynamics and MM/PBSA analyses identified Apigenin-7-*O*-glucuronide as the most energetically favourable ligand. Furthermore, its favourable binding free energies toward both CDR1 and GSC1 provide additional computational evidence supporting its potential as a putative dual-target antifungal lead for further experimental investigation.

### 3.7 HR-LCMS characterization of *Eucalyptus camaldulensis* extract

To provide analytical support for the computational predictions, the hydroethanolic extract of *Eucalyptus camaldulensis* was analysed by high resolution liquid chromatography-mass spectrometry (HR-LCMS). The total ion chromatogram showed the presence of many phytochemical constituents indicating the complex chemical composition of the crude extract (**Supplementary Figure 8**).

Accurate mass analysis provided the putative identification of Apigenin-7-O-glucuronide, the top phytochemical prioritised by molecular docking, molecular dynamics simulations, MM/PBSA analysis, and cross-target screening. The compound has a molecular formula C_21_H_18_O_11_ and monoisotopic molecular weight of 446.08 Da. HR-LCMS analysis showed a strong protonated molecular ion [M+H] + at m/z 447.31 in good agreement with the theoretical protonated mass of Apigenin-7-O-glucuronide. The chromatographic retention profile, high mass accuracy, and database matching provided putative evidence for the presence of this phytochemical in the extract of *Eucalyptus camaldulensis.*

HR-LCMS analysis showed the presence of other several phytochemical constituents apart from Apigenin-7-O-glucuronide, showing the chemical diversity of the crude extract. As with crude plant extract, the observed antifungal activity is likely due to the combined effects of more than one metabolite. However, the putative detection of Apigenin-7-O-glucuronide provides analytical support for the computational screening strategy and forms an important bridge between *in silico* lead identification and subsequent biological evaluation.

The HR-LCMS results altogether supports the presence of the computationally prioritised phytochemical in the hydroethanolic extract of *Eucalyptus camaldulensis* and thus supports the choice of the extract for further *in vitro* antifungal evaluation.

### 3.8 Initial *In Vitro* evidence supports the computational predictions

To obtain preliminary experimental evidence supporting the computational findings, the antifungal activity of the hydroethanolic extract of *Eucalyptus camaldulensis*, in which Apigenin-7-*O*-glucuronide was putatively identified by HR-LCMS, was evaluated against both the reference strain *Candida albicans* ATCC 10231 and environmentally isolated drug-resistant *Candida* and emerging pathogenic yeasts.

#### 3.8.1 Antifungal drug susceptibility of environmental *Candida* and emerging pathogenic yeast isolates

The antifungal resistance profile of the environmental isolates was determined by antifungal susceptibility testing using the HiMedia antifungal susceptibility kit containing Caspofungin, Flucytosine, Fluconazole and Itraconazole. The tested isolates were previously isolated from environmental samples and molecular identification was done using 18S rRNA gene sequencing and the nucleotide sequences were submitted to NCBI GenBank database (Mundhe and Rajkumari, 2026).

**Supplementary Figure 9** shows the differential susceptibility profiles of the environmental *Candida* and emerging pathogenic yeast isolates to the tested antifungal agents. Several isolates showed reduced susceptibility or resistance to one or more of the azole antifungals, particularly fluconazole and itraconazole. Susceptibility to caspofungin and flucytosine was variable among isolates. The resistance profiles obtained highlight the rise of antifungal resistance in environmental yeasts and the importance of alternative therapeutic options against resistance-related mechanisms such as the CDR1 efflux transporter.

These results gave the biological rationale to test the hydroethanolic extract of *Eucalyptus camaldulensis* containing the putative identified Apigenin-7-O-glucuronide against both the reference strain and the characterized environmental isolates.

#### 3.8.2 Qualitative antifungal activity of *Eucalyptus camaldulensis* extract

The hydroethanolic extract of *Eucalyptus camaldulensis*, in which Apigenin-7-*O*-glucuronide was putatively identified by HR-LCMS analysis, exhibited concentration-dependent antifungal activity against the reference strain *Candida albicans* ATCC 10231 as well as the selected environmental *Candida* and emerging pathogenic yeast isolates (**Figure 11**). Agar well diffusion assays demonstrated clear inhibition zones surrounding the wells containing the plant extract, indicating effective suppression of fungal growth.

**Figure 11:**
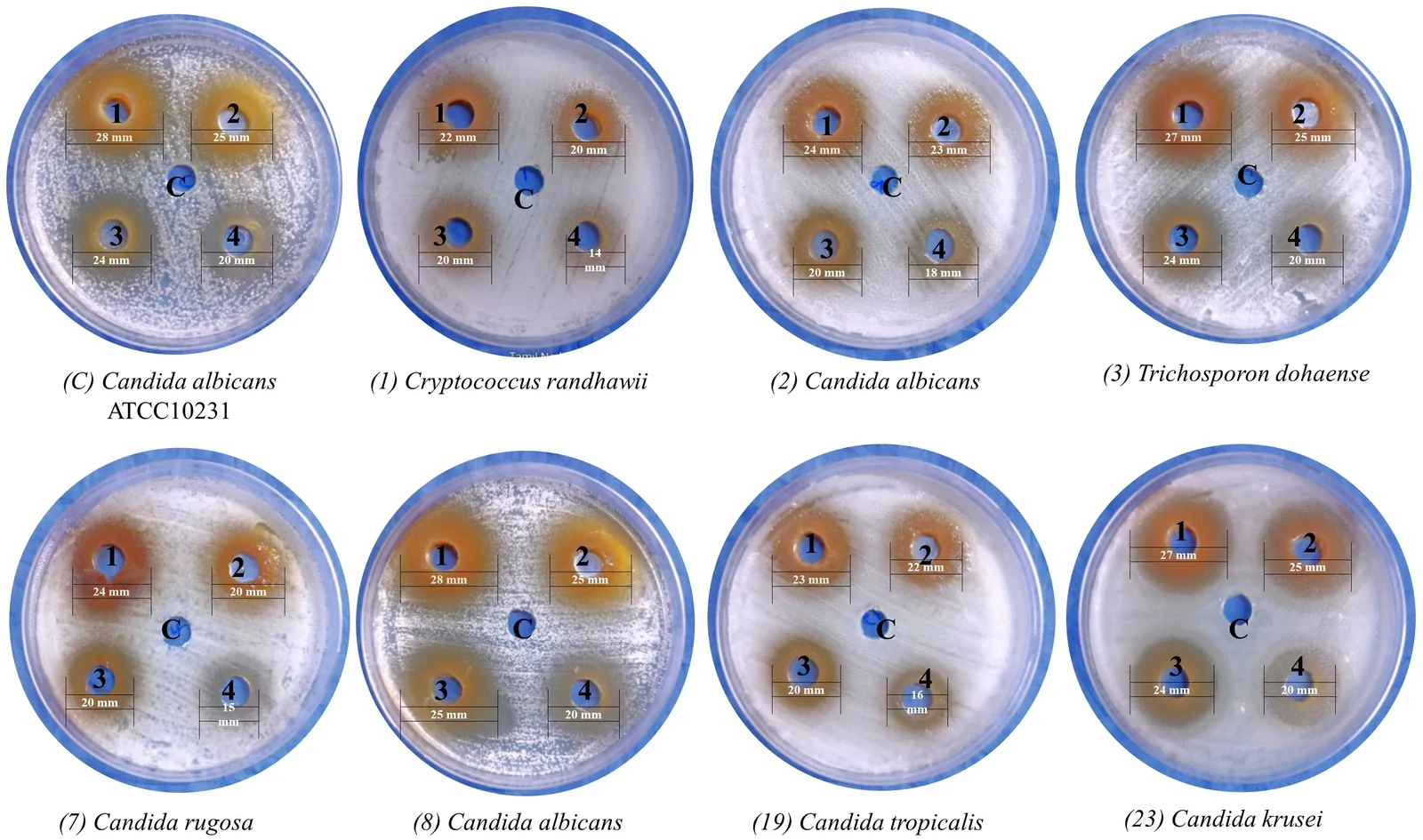
Qualitative antifungal activity of the hydroethanolic extract of *Eucalyptus camaldulensis*. Representative agar well diffusion assay showing the concentration-dependent antifungal activity of the hydroethanolic extract of *Eucalyptus camaldulensis*, containing putatively identified Apigenin-7-*O*-glucuronide, against *Candida albicans* ATCC 10231 and selected environmental yeast isolates. Well 1 contained 100 mg/mL of plant extract, well 2–50 mg/mL, well 3–25 mg/mL, well 4 contained 12.5 mg/mL and central well served as dilution solvent control.

At the highest tested concentration (100 mg/mL), the extract produced the largest inhibition zones against all tested isolates. The inhibition zones against *Candida albicans* ATCC 10231 (28 mm at 100 mg/mL) were reduced to 25 mm, 24 mm and 20 mm after successive two-fold dilutions (50, 25 and 12.5 mg/mL respectively). Similar concentration-dependent inhibition was seen for the environmental isolates. *Cryptococcus randhawii* showed inhibition zones of 22 mm, 20 mm, 20 mm and 14 mm and environmental *Candida albicans* isolate showed inhibition zones of 24 mm, 23 mm, 20 mm and 18 mm. *Trichosporon dohaense* showed relatively higher sensitivity with inhibition zones of 27, 25, 24 and 20 mm, closely resembled the response of the reference strain. The second environmental isolates *Candida albicans*, *Candida tropicalis* and *Candida krusei* also showed zones of inhibition of 20–28 mm at higher extract concentrations and 14–20 mm at the lowest effective concentration.

The inhibition zones showed a tendency to reduce steadily with the reduction in extract concentration, showing a clear dose-dependent antifungal effect. Although the crude extract contains multiple phytochemicals that may collectively contribute to the observed activity, the putative presence of Apigenin-7-*O*-glucuronide, identified computationally as the highest-ranked and only dual-target (CDR1–GSC1) phytochemical lead, provides initial biological evidence supporting the phytochemical-guided selection of *Eucalyptus camaldulensis* for antifungal evaluation.

#### 3.8.3 Quantitative antifungal activity

The antifungal activity of the hydroethanolic extract of *Eucalyptus camaldulensis*, containing putatively identified Apigenin-7-*O*-glucuronide, was further evaluated by the broth microdilution method to determine the minimum inhibitory concentration (MIC) and minimum fungicidal concentration (MFC) against *Candida albicans* ATCC 10231 and the selected environmental *Candida* and emerging pathogenic yeast isolates (**Supplementary Figure 10**).

Complete inhibition of visible fungal growth was observed in all tested isolates at extract concentrations ranging from 50 mg/mL to 1.56 mg/mL. In contrast, visible fungal growth reappeared at the subsequent two-fold dilution (0.78 mg/mL), indicating that the MIC of the hydroethanolic extract was 1.56 mg/mL for all the tested organisms.

To distinguish fungistatic from fungicidal activity, aliquots from the three consecutive growth-free wells (1.56, 3.125, and 6.25 mg/mL) were subcultured onto Sabouraud Dextrose Agar (SDA) plates. Growth was observed from cultures corresponding to the 1.56 mg/mL and 3.125 mg/mL wells, whereas no fungal growth was detected from the 6.25 mg/mL well following incubation. Accordingly, the minimum fungicidal concentration (MFC) of the extract was determined to be 6.25 mg/mL (**Table 4**).

**Table 4:** Initial *in vitro* antifungal activity of the hydroethanolic *Eucalyptus camaldulensis* extract containing the putatively identified computational lead phytochemical Apigenin-7-*O*-glucuronide against *Candida albicans* and environmental *Candida* and emerging pathogenic yeast isolates.

Sr. No.	Test organism	Zone of inhibition (mm) at 100 mg/mL	Zone of inhibition (mm) at 50 mg/mL	Zone of inhibition (mm) at 25 mg/mL	Zone of inhibition (mm) at 12.50 mg/mL	MIC (mg/mL)	MFC (mg/mL)
1	*Candida albicans* ATCC 10231	28	25	24	20	1.56	6.25
2	*Cryptococcus randhawii*	22	20	20	14	1.56	6.25
3	*Candida albicans* (Environmental isolate)	24	23	20	18	1.56	6.25
4	*Trichosporon dohaense*	27	25	24	20	1.56	6.25
5	*Candida rugosa*	24	22	20	18	1.56	6.25
6	*Candida albicans* (Environmental isolate II)	28	24	22	20	1.56	6.25
7	*Candida tropicalis*	26	24	22	18	1.56	6.25
8	*Candida krusei*	24	22	20	16	1.56	6.25

The broth microdilution assay therefore confirmed the potent antifungal activity of the hydroethanolic *Eucalyptus camaldulensis* extract against both the reference strain and the environmentally derived drug-resistant *Candida* and emerging pathogenic yeast isolates. When interpreted together with the agar well diffusion assay, HR-LCMS characterization, and computational analyses, these findings provide initial *in vitro* evidence supporting the antifungal potential of *Eucalyptus camaldulensis*. Although the observed activity cannot be attributed exclusively to Apigenin-7-*O*-glucuronide, due to the complex phytochemical composition of the crude extract, the putative presence of this computationally prioritized dual-target phytochemical provides a strong rationale for its future isolation and direct biological evaluation.

## 4 Discussion

The rapid emergence of antifungal resistance among *Candida* species has significantly reduced the therapeutic efficacy of conventional antifungal agents, particularly azoles, which necessitates alternative treatments targeting resistance-associated pathways (Prasad and Rawal, 2014; Perfect, 2017; Fisher et al., 2022). At the molecular scale, the ATP-binding cassette transporter CDR1 plays an essential role in the multidrug resistance mechanisms, which are responsible for the active efflux of antifungal drugs from fungal cells thus reducing the intracellular drug accumulation and therapeutic efficacy. Thus, the inhibition of CDR1 is a potential strategy to restore antifungal susceptibility. The present study involved large-scale virtual screening of 17967 phytochemicals from IMPPAT database and identified multiple putative CDR1 inhibitors. This study emphasises the necessity of combining natural product libraries with structure-based computational approaches for antifungal lead discovery.

A major outcome of this study was the consistent identification of Apigenin-7-O-glucuronide as the most promising lead throughout the integrated computational workflow. While the molecular docking score of Shihulimonin A was the highest on CDR1 initially, molecular dynamics simulation and MM/PBSA binding free-energy calculations improved the ranking of the compounds and identified the most favourable overall binding energetics for Apigenin-7-O-glucuronide. This observation points to the limitations of using docking scores alone for lead prioritisation and the need to combine molecular dynamics simulations and free-energy calculations to evaluate the dynamic stability of protein–ligand complexes (Kitchen et al., 2004; Genheden and Ryde, 2015). Similar observations were also reported in previous computational studies where stable hydrophobic and electrostatic interactions played a more prominent role in ligand binding than hydrogen-bond occupancy alone (Kumari et al., 2014).

Interestingly, despite exhibiting the highest docking score and a greater number of intermolecular hydrogen bonds during molecular dynamics simulations, Shihulimonin A yielded an unfavourable MM/PBSA binding free energy (+1.31 kJ/mol). This discrepancy indicates that favourable docking poses and increased hydrogen-bond occupancy alone do not necessarily translate into thermodynamically stable binding. MM/PBSA energy decomposition showed that the favourable van der Waals and electrostatic interactions were largely offset by a substantial polar solvation (desolvation) penalty, resulting in an overall unfavourable binding free energy. Thus, although Shihulimonin A maintained intermolecular interactions throughout the simulation, the energetic cost associated with solvent reorganization outweighed these favourable contacts. In contrast, Apigenin-7-*O*-glucuronide achieved a more favourable balance between intermolecular interactions and solvation energy, resulting in a substantially lower binding free energy despite forming fewer hydrogen bonds. These findings highlight that docking scores and hydrogen-bond occupancy should be interpreted together with molecular dynamics and binding free-energy analyses when prioritizing lead compounds. Although hydrogen bond lifetime analysis and additional conformational analyses could provide further mechanistic insights, they were beyond the scope of the present study.

Another interesting observation was that Apigenin-7-O-glucuronide was the only shortlisted phytochemical that showed favourable interactions with both CDR1 and GSC1 in systematic cross-target screening of all the ten prioritised CDR1 leads. CDR1 mediates antifungal drug efflux while GSC1 catalyses β-(1,3)-glucan biosynthesis. Thus, simultaneous modulation of two resistance-associated targets may confer therapeutic advantage by disrupting intracellular drug efflux as well as fungal cell-wall synthesis (Prasad and Rawal, 2014; Perlin, 2015a). Although this dual- target activity needs to be validated by direct experiments with purified compounds and target-specific assays, the combination of docking, molecular dynamics and MM/PBSA analyses provides a strong computational rationale to explore Apigenin-7-O-glucuronide as a promising multitarget antifungal lead. These polypharmacological strategies are increasingly getting recognised as effective approaches to combat antimicrobial resistance as the simultaneous modulation of multiple pathways may reduce the chances of the development of resistance (Anighoro et al., 2014; Proschak et al., 2019).

Qualitative and quantitative antifungal assays revealed concentration- dependent inhibition of *Candida albicans* ATCC 10231 and genetically characterised environmental *Candida* and emerging pathogenic yeast isolates. The extract showed a MIC of 1.56 mg/mL and an MFC of 6.25 mg/mL, thus providing initial *in vitro* evidence supporting the computational predictions. However, since the biological assays were conducted with a crude plant extract, the observed antifungal activity cannot be exclusively attributed to Apigenin-7-O-glucuronide. Other phytochemical constituents may also contribute through additive or synergistic interactions, a phenomenon often reported for medicinal plant extracts (Wagner and Ulrich-Merzenich, 2009).

One of the major strengths of the present study is the use of environmental *Candida* and emerging pathogenic yeast isolates, identified earlier by 18S rRNA gene sequencing and deposited in NCBI GenBank database (Mundhe and Rajkumari, 2026), rather than using only laboratory reference strains. Inclusion of isolates with variable susceptibility to Caspofungin, Flucytosine, Fluconazole and Itraconazole increases the biological relevance of the study and indicates potential applicability of phytochemical based antifungal agents against various environmental opportunistic yeasts.

The integrated workflow described here greatly increases confidence in the identified lead phytochemical, but several limitations should be noted. Although the computational analyses consistently prioritized Apigenin-7-O-glucuronide as the most promising multitarget lead, the experimental findings should not be interpreted as direct validation of this phytochemical. The biological evaluation was performed using crude hydroethanolic extracts of *Eucalyptus camaldulensis*, for which HR-LCMS provided only putative evidence for the presence of Apigenin-7-O-glucuronide. As crude extracts contain numerous phytochemical constituents, the observed antifungal activity may result from synergistic or additive effects rather than the activity of a single compound (Atanasov et al., 2021). Therefore, the biological findings should be regarded as preliminary support for the computational screening strategy rather than definitive evidence of the antifungal activity of Apigenin-7-O-glucuronide. According to the Metabolomics Standards Initiative (MSI) framework, the present HR-LCMS evidence corresponds to a putative Level 3 identification. Future studies should focus on the isolation of the phytochemical followed by definitive structural confirmation using MS/MS fragmentation and NMR spectroscopy (Wolfender et al., 2019). Subsequent studies should include target-specific validation through CDR1 efflux inhibition assays, GSC1 inhibition studies, gene expression analyses, and comparative evaluation with established CDR1 inhibitors such as Beauvericin to experimentally validate the predicted dual-target mechanism (Prasad and Rawal, 2014; Cowen et al., 2015; Anighoro et al., 2014).

Taken together, the present study shows that the combination of large-scale virtual screening, cross-target analysis, molecular dynamics simulation, MM/PBSA binding free-energy calculations, HR-LCMS phytochemical characterisation, and initial *in vitro* antifungal evaluation provides a robust and cost-effective workflow for natural-product-based antifungal drug discovery (Atanasov et al., 2021). The concordance between the computational prioritization and the preliminary antifungal activity observed for the crude extract provides biological support for the phytochemical-guided screening strategy and identifies Apigenin-7-O-glucuronide as a putative dual-target lead for further investigation.

## 5 Conclusion

The present study developed an integrated computational–experimental workflow for the identification and prioritization of phytochemical leads targeting antifungal resistance-associated proteins in *Candida albicans*. Large-scale virtual screening of 17,967 phytochemicals from the IMPPAT database, followed by molecular docking, ADMET evaluation, molecular dynamics simulations, and MM/PBSA binding free-energy analyses, identified multiple CDR1-binding phytochemicals. Among these, Apigenin-7-*O*-glucuronide was prioritized as a putative dual-target antifungal lead based on its favourable binding interactions and dynamic stability with both CDR1 and GSC1.

HR-LCMS characterization of the hydroethanolic extract of *Eucalyptus camaldulensis* provided putative evidence for the presence of Apigenin-7-*O*-glucuronide, while qualitative and quantitative antifungal assays demonstrated reproducible antifungal activity of the crude extract against *Candida albicans* ATCC 10231 and environmentally derived drug-resistant *Candida* and emerging pathogenic yeast isolates. As these biological evaluations were performed using a crude plant extract, the observed antifungal activity cannot be exclusively attributed to Apigenin-7-*O*-glucuronide and may instead reflect the combined effects of multiple phytochemical constituents. Nevertheless, these findings provide preliminary biological evidence supporting the computational predictions and the phytochemical-guided screening strategy.

Future studies should focus on the isolation and structural confirmation of Apigenin-7-*O*-glucuronide, followed by target-specific biochemical validation, including CDR1 efflux inhibition assays, GSC1 inhibition studies, gene expression analyses, and *in vivo* investigations to experimentally validate its predicted dual-target activity. Overall, this study demonstrates that integrating large-scale virtual screening with computational refinement and preliminary biological evaluation provides a robust framework for accelerating the discovery and prioritization of natural product-based antifungal leads against multidrug-resistant fungal pathogens.

## Data Availability

The original contributions presented in the study are included in the article/**Supplementary Material**. Further inquiries can be directed to the corresponding authors.
