# Supplementary material for: Large-scale virtual screening of 17,967 IMPPAT phytochemicals identifies multiple CDR1 binders and reveals Apigenin-7-O-glucuronide as a putative CDR1-GSC1 dual-target lead against Candida albicans with initial in-vitro evidence
Source: Front Cell Infect Microbiol. 2026 Sep 14;16:1940083. doi: 10.3389/fcimb.2026.1940083 (PMC13616775; doi:10.3389/fcimb.2026.1940083)
Supplement: Supplementary file 1 [file DataSheet1.pdf]

Supplementary figure captions:

Figure S1. Workflow of ultrasound-assisted phytochemical extraction of *Eucalyptus camaldulensis*

Figure S2. Three-dimensional structures of *Candida albicans* target proteins employed for virtual screening. The AlphaFold-predicted structures of CDR1, CDR2, ERG1, ERG6, ERG11, and GSC1 represent key proteins involved in antifungal drug efflux, ergosterol biosynthesis, and fungal cell wall synthesis (Mundhe and Rajkumari, 2025a).

Figure S3. Ramachandran plots of AlphaFold-predicted *Candida albicans* target proteins. Ramachandran plots generated from PROCHECK-validated protein structures of CDR1, CDR2, ERG1, ERG6, ERG11, and GSC1 showing the distribution of backbone φ (phi) and ψ (psi) torsional angles.

Figure S4. RMSF analysis of CDR1–phytochemical complexes during molecular dynamics simulations.
Note: Root mean square fluctuation (RMSF) profiles of amino acid residues of native CDR1 (black) and ligand bound CDR1 complexes (red) obtained from 100 ns molecular dynamics simulations. RMSF values represent the flexibility of each residue and structural fluctuations after binding of phytochemicals.

Figure S5. Radius of gyration (Rg) analysis of CDR1–phytochemical complexes.
Note: Radius of gyration (Rg) trajectories of native CDR1 (black) and CDR1 complexes bound with ligands (red) during the 100 ns molecular dynamics simulations. The Rg analysis was used to evaluate the changes in the overall protein compactness upon ligand binding.

Figure S6. Solvent-accessible surface area (SASA) analysis of CDR1–phytochemical complexes.
Note: Solvent accessible surface area (SASA) trajectories of native CDR1 (black) and ligand bound CDR1 complexes (red) during the 100 ns molecular dynamics simulations. The SASA analysis considers the change in solvent exposure as a consequence of the conformational change induced by the ligands.

Figure S7. Principal component analysis (PCA) of CDR1 complexes with Rhynchosin and Undulatoside A.

Figure S8. HR-LCMS characterization of the hydroethanolic extract of *Eucalyptus camaldulensis*.
Note: Representative high-resolution mass spectrum of the hydroethanolic extract of *Eucalyptus camaldulensis*. **Apigenin-7-*O*-glucuronide** (molecular formula $\mathbf{C_{21}H_{18}O_{11}}$; monoisotopic molecular weight **446.08 Da**) was **putatively identified** based on detection of the protonated molecular ion $\mathbf{[M+H]^+}$ **at *m/z* 447.31**.

Figure S9. Antifungal susceptibility profiles of wild *Candida* and emerging pathogenic yeast isolates.

Note: Antifungal susceptibility patterns of molecularly characterized environmental *Candida* and emerging pathogenic yeast isolates against **Caspofungin, Flucytosine, Fluconazole, and Itraconazole** using the HiMedia antifungal susceptibility kit. The isolates were previously identified by **18S rRNA gene sequencing**, and the corresponding nucleotide sequences were deposited in the **NCBI GenBank** database (Mundhe and Rajkumari, 2026). Variations in susceptibility among the isolates demonstrate the occurrence of antifungal resistance and provide the rationale for evaluating phytochemical-based antifungal agents.

Figure S10. Quantitative antifungal activity of the hydroethanolic extract of *Eucalyptus camaldulensis*.

Note: Representative broth microdilution assay used to determine the **minimum inhibitory concentration (MIC)** and **minimum fungicidal concentration (MFC)** of the hydroethanolic extract of *Eucalyptus camaldulensis* against *Candida albicans* ATCC 10231 and selected environmental *Candida* and emerging pathogenic yeast isolates. Complete inhibition of visible growth was observed up to **1.56 mg/mL** (MIC), while the **MFC was determined to be 6.25 mg/mL** following subculture of growth-free wells onto Sabouraud Dextrose Agar.
